# Bursting in cerebellar stellate cells induced by pharmacological agents: Non-sequential spike adding

**DOI:** 10.1371/journal.pcbi.1008463

**Published:** 2020-12-14

**Authors:** Saeed Farjami, Ryan P. D. Alexander, Derek Bowie, Anmar Khadra

**Affiliations:** 1 Department of Physiology, McGill University, Montréal, QC, Canada; 2 Department of Pharmacology and Therapeutics, McGill University, Montréal, QC, Canada; University of Pittsburgh, UNITED STATES

## Abstract

Cerebellar stellate cells (CSCs) are spontaneously active, tonically firing (5-30 Hz), inhibitory interneurons that synapse onto Purkinje cells. We previously analyzed the excitability properties of CSCs, focusing on four key features: type I excitability, non-monotonic first-spike latency, switching in responsiveness and runup (i.e., temporal increase in excitability during whole-cell configuration). In this study, we extend this analysis by using whole-cell configuration to show that these neurons can also burst when treated with certain pharmacological agents separately or jointly. Indeed, treatment with 4-Aminopyridine (4-AP), a partial blocker of delayed rectifier and A-type K^+^ channels, at low doses induces a bursting profile in CSCs significantly different than that produced at high doses or when it is applied at low doses but with cadmium (Cd^2+^), a blocker of high voltage-activated (HVA) Ca^2+^ channels. By expanding a previously revised Hodgkin–Huxley type model, through the inclusion of Ca^2+^-activated K^+^ (K(Ca)) and HVA currents, we explain how these bursts are generated and what their underlying dynamics are. Specifically, we demonstrate that the expanded model preserves the four excitability features of CSCs, as well as captures their bursting patterns induced by 4-AP and Cd^2+^. Model investigation reveals that 4-AP is potentiating HVA, inducing square-wave bursting at low doses and pseudo-plateau bursting at high doses, whereas Cd^2+^ is potentiating K(Ca), inducing pseudo-plateau bursting when applied in combination with low doses of 4-AP. Using bifurcation analysis, we show that spike adding in square-wave bursts is non-sequential when gradually changing HVA and K(Ca) maximum conductances, delayed Hopf is responsible for generating the plateau segment within the active phase of pseudo-plateau bursts, and bursting can become “chaotic” when HVA and K(Ca) maximum conductances are made low and high, respectively. These results highlight the secondary effects of the drugs applied and suggest that CSCs have all the ingredients needed for bursting.

## Author summary

Excitable cells, including neurons, fire action potentials (APs) in their membrane voltage that allow them to communicate with each other and to serve certain physiological purposes. They do so either tonically by firing APs periodically, or episodically by repeatedly firing clusters of APs (called bursts) separated by quiescent periods. Each one of those firing patterns can be neuron-specific and dependent on synaptic inputs and/or their physiological environment. Cerebellar stellate cells (CSCs) that synapse onto Purkinje cells, the sole output of the cerebellum responsible for motor control, are spontaneously active inhibitory interneurons that fire APs tonically. We previously studied the excitability properties of these neurons and showed that they possess several important key features, including type I excitability, runup, non-monotonic first spike latency and switching in responsiveness. In this study, we show that CSCs can also exhibit two modes of burst firing, called square-wave and pseudo-plateau, when treated with certain pharmacological agents. Using bifurcation theory, we demonstrate that spike adding in the square-wave burst is non-sequential, changing by several spikes when certain conductances are altered gradually. This study thus sheds lights onto the overall effects of the pharmacological agents and highlights the ability of CSCs to burst in certain biological conditions.

## Introduction

Cerebellar stellate cells (CSCs) are small, fast-firing inhibitory interneurons located in the superficial cerebellar cortex [1, 2]. Due to their high input resistance and electrically compact nature, they are ideal mediators of reliable feedforward inhibition onto their synaptic partners [3]. Their position in the cerebellar cortical circuit allows them to integrate glutamatergic input from both parallel and climbing fibers [4, 5], along with neuromodulatory signals via aminergic afferents [6, 7], to provide efficient GABAergic innervation to neighboring interneurons, as well as to the principal output Purkinje cells [8]. The level of CSC activity has been demonstrated to regulate not only simple spiking in Purkinje cells [9], but also directly impact Ca^2+^ entry in their dendritic arbors, having dramatic control over synaptic plasticity and motor learning [10]. CSC firing behavior is dynamic and highly variable, controlled by a complex interplay of many voltage-dependent conductances [11–13] and unique passive membrane properties [14, 15]. These cells generally exhibit tonic firing of up to 100 Hz (with sufficient depolarizing input) [10], and have not been observed to oscillate or burst in vivo.

We have previously analyzed the main features of CSCs, including type I excitability, runup (i.e., temporal increase in excitability) non-monotonic first-spike latency and switching in responsiveness when a pair of inhibitory and excitatory post-synaptic inputs are applied [16, 17]; a revised Hodgkin–Huxley type model was used to accomplish this. The model was originally developed in [18], but later reparametrized to capture more accurately the cycles of action potentials (APs) in $\left(V,\dot{V}\right)$-plane during both pre- and post-runup conditions [13]. In addition to the standard ionic currents in the Hodgkin and Huxley formalism [19], namely, sodium (Na^+^), delayed rectifier potassium (K^+^) and leak currents, the model also included the A-type K^+^ and T-type calcium (Ca^2+^) currents. The model produced tonic firing activity during both pre- and post-runup with a higher frequency and lower firing threshold in the later compared to the former [13]. It provided important insights into the underlying dynamics of the four features listed above.

One interesting aspect of many neurophysiological systems is their ability to exhibit burst firing activity in their membrane voltage characterized by the emergence of repetitive clusters of APs separated by quiescent periods [20]. It has been suggested that such bursts are more efficient at releasing neurotransmitters and/or hormones as well as better at preventing receptors from desensitization compared to tonic firing [21]. Cerebellar Purkinje cells that receive synaptic inputs from CSCs, for example, are spontaneously active neurons that tonically fire, but their firing switches to bursting upon receiving inhibitory or excitability inputs, exhibiting different modes of burst firing [22]. Although CSCs do not spontaneously burst under normal conditions, when no pharmacological agents are applied, we show here that under various pharmacological conditions, they do exhibit two distinct modes of burst firing when bathed in 4-Aminopyridine (4-AP) alone or in combination with cadmium (Cd^2+^). The drug 4-AP is known to partially block voltage-gated K^+^ channels (such as the A-type and delayed rectifier K^+^ channels) [23], whereas Cd^2+^ is known to block voltage-gated Ca^2+^ channels [24]. With such effects in mind, one would expect the 4-AP to increase excitability of CSCs whereas Cd^2+^ to decrease it. Based on the results presented here, it seems that the effects of these drugs are significantly more complex. To further investigate the effects of these two drugs and determine how the two modes of burst firing are generated, we have expanded in this study our previously revised Hodgkin–Huxley type model by including two additional ionic currents (namely, high voltage-activated (HVA) Ca^2+^ and Ca^2+^-activated K^+^ (K(Ca)) currents) and cytosolic Ca^2+^ dynamics ([Ca^2+^]_*i*_) to the model. With this new formalism, we have managed to identify which current conductances are affected with the two drugs and elucidate their intricate role in inducing bursting.

Bursting periodic orbits (BPOs) encode several time scales ranging from slow to fast, allowing neurons (and other excitable cells such as *β* cells) to exhibit such patterns of activity. Using slow-fast analysis, first introduced in [25, 26], BPOs were classified based on their underlying dynamics [26–30], and later expanded in a recent study using canard theory [31]. The application of slow-fast analysis has been extended to many Hodgkin–Huxley type models describing the bursting dynamics of many neurophysiological and endocrine systems, including for example inner hair cells [32], embryonic pre-BötC neuron [33] and pancreatic *β* cells [34]. It provides a tool to determine how different time scales interact and how they are responsible in generating the bursting activity observed.

In this study, we employ both bifurcation and slow-fast analysis to study the dynamics of the expanded CSC Hodgkin–Huxley model. The bifurcation analysis is performed to see how key current conductances alter the electrical properties of the cell (including for examples the active phase duration and number of spikes in a BPO), while slow-fast analysis is conducted to determine what factors govern the switching between the active and silent phases in the two bursting modes. Our results reveal that the topology of bifurcation diagram associated with the two modes of bursting mimic those that produce square-wave (fold/homoclinic) and pseudo-plateau bursting (fold/sub-Hopf), respectively. The number of spikes in the square-wave BPOs obtained by this model appears, however, to be very sensitive to parameter perturbations in a manner similar to what have been previously seen in a bursting model of leech heart interneurons [35].

The number of spikes in the active phase of a BPO is one of the most important and probably obvious features of a BPO. From mathematical viewpoint, the underlying mechanism for generating a certain number of oscillations (spikes) and altering their number could be an interesting problem that has biological implications. This change in the number of spikes happens through usually complex mechanisms in an exponentially small parameter interval (depending on the time-scale difference), is sensitive to noise and could be chaotic [35]. The entire process of generating new spikes and the underlying mechanism(s) involved is called spike adding. Previously, spike adding phenomena have been vastly studied in systems with different time scales [36–40]. Although, there are a number of similarities between these studies and the one presented here (e.g., the presence of infinite number of isolas delimited by period-doubling and saddle-node bifurcations of periodic orbits [36, 40–42]), they still differ by the fact that spike adding in the previous studies is sequential (increasing by one spike at a time when a specific parameter is varied), but not so in the square-wave bursting mode presented in this study (i.e., non-sequential), a feature that has not been reported before. It has been shown that for three-dimensional systems with two slow variables, there exist small (secondary) isolas between two large (primary) ones in which the number of spikes per burst alternates between the number of spikes of the two neighboring primary ones [36, 38, 43]. In this case, the number of spikes added in the primary isolas is one. Furthermore, another interesting aspect of these previous studies is that the number of spikes keep increasing/decreasing when a given parameter in the system is varied. Interestingly, these previously observed characteristics of spike adding are not valid for the expanded Hodgkin–Huxley type CSC model presented here. Indeed, we find that spike adding during square-wave busting generating by this model is non-sequential and that the primary and secondary isolas responsible for producing the BPOs appear to be random. We provide in this study a hypothesis as to why we observe such outcomes.

## Methods

### Slice preparation and electrophysiology

Slice preparation and whole-cell electrophysiological recordings were performed as previously described (see [13]). Briefly, 300 *μ*M sagittal slices of wild-type C57BL/6 mouse cerebellar vermis were obtained by vibrating microtome in ice-cold sucrose-based cutting solution. Slices were incubated at room temperature for 1 hour before recording. Whole-cell patch clamp recordings were made while tissue was perfused with oxygenated, room temperature ACSF on CSCs located in the upper one third of the molecular layer. Note that 2 and 20 mM 4-AP and 300 *μ*M cadmium chloride (CdCl_2_) were dissolved in ACSF prior to recording in some experiments. All chemicals were obtained from Sigma Aldrich.

### Mathematical model

The original Hodgkin–Huxley type model developed in [18] and revised in [13] is not able to generate bursting activities due to the absence of Ca^2+^-activated K^+^ (K(Ca)) current from the model (results not shown). Since K(Ca) channels (e.g., SK and BK channels) and high voltage-activated (HVA) Ca^2+^ channels are both expressed in CSCs [44, 45], the model is expanded by including the currents produced by both of these channels. Since K(Ca) is Ca^2+^-dependent, the dynamics of [Ca^2+^]_*i*_ (denoted by *Ca*) is also included in the model. The resulting expanded system (referred to hereafter as the “full system”) is thus given by

$$\left\{ \begin{array}{cc} C\dot{V} & =I_{\text{app}}-I_{\text{Na}}-I_{K}-I_{L}-I_{A}-I_{T}-I_{K(\text{Ca})}-I_{\text{HVA}}\\ \dot{x} & =\left(x_{\infty }-x\right)/\tau_{x},\;x=h,\;n,\;n_{A},\;h_{A},\;h_{T},\;m_{\text{HVA}}\\ \dot{Ca} & =-\varepsilon (\alpha \left(I_{T}+I_{\text{HVA}}\right)+kCa), \end{array}\right.$$ (1)

where *C* is the membrane capacitance and *I*_app_ is the applied current. The ionic currents included in the model are

$$\begin{array}{c} \begin{array}{cc} I_{\text{Na}} & =g_{\text{Na}}\;m_{\infty }^{3}\;h\;\left(V-E_{\text{Na}}\right)\\ I_{K} & =g_{K}\;n^{4}\;\left(V-E_{K}\right)\\ I_{L} & =g_{L}\;\left(V-E_{L}\right)\\ I_{A} & =g_{A}\;n_{A}\;h_{A}\;\left(V-E_{K}\right)\\ I_{T} & =g_{T}\;m_{T,\infty }\;h_{T}\;\left(V-E_{\text{Ca}}\right)\\ I_{\text{HVA}} & =g_{\text{HVA}}\;m_{\text{HVA}}\;\left(V-E_{\text{Ca}}\right)\\ I_{K(\text{Ca})} & =g_{K(\text{Ca})}\;\left(Ca^{5}/\left(k_{Ca}^{5}+Ca^{5}\right)\right)\;\left(V-E_{K}\right), \end{array} \end{array}$$ (2)

where *g*_*θ*_ and *E*_*θ*_, *θ* = Na, K, L, A, T, HVA, K(Ca) are the maximum conductance and reversal potential of each ionic current, respectively. Notice here that, according to (2), the activation variables of *I*_Na_ and *I*_A_ are assumed to be fast (i.e., at steady state), and thus not included in the variable *x* in (1). The dynamics of the gating variables of the voltage-activated ionic currents *I*_*η*_, (*η* = Na, K, L, A, T, HVA) are dictated by state variables *x*, where *x*_∞_ and *τ*_*x*_ denote the steady state and time constant for (in)activation, respectively. The steady state (in)activation functions are monotonic functions, given by

$$\begin{array}{c} x_{\infty }=\frac{1}{1+e^{-\left(V-v_{x}\right)/s_{x}}},\;x=m,\;h,\;n,\;n_{A},\;h_{A},\;n_{T},\;h_{T},\;h_{\text{HVA}}, \end{array}$$

whereas the time constants for Na^+^ inactivation is given by

$$\begin{array}{c} \tau_{h}=y_{0}+\frac{2Aw}{4\pi {\left(V-V_{c}\right)}^{2}+w^{2}} \end{array}$$

and for K^+^ activation by

$$\begin{array}{c} \tau_{n}=\frac{6}{1+e^{(V+23)/15}}. \end{array}$$

As suggested above, the activation of *I*_Na_ (*m*_∞_) and *I*_T_ (*m*_T,*∞*_) are assumed to be fast and set to steady state. The full model, given by (1) and (2), has been reparametrized in a manner similar to the method used in [16, 17] to capture the profile of the AP-cycle in the $\left(V,\dot{V}\right)$-plane. The resulting parameters are kept fixed throughout the paper, unless otherwise stated. A full list of these parameters is provided in Tables 1 and 2 under control condition.

**Table 1:** Parameter values of the voltage-dependent ionic currents included in Eq (1). Whenever two values are provided for a single parameter, the first corresponds to pre-runup (*t* = 0 min after patch clamping), while the second (between parentheses) corresponds to post-runup (*t* = 25 min after patch clamping).

	Activation			Inactivation		
	*v*_*x*_ (mV)	*s*_*x*_ (mV)	*τ*_*x*_ (ms)	*v*_*x*_ (mV)	*s*_*x*_ (mV)	*τ*_*x*_ (ms)
*I*_Na_	−37(−45.2)	+ 3(+ 2.3)	-	−40(−51.5)	−4	*τ*_*h*_(*V*)
*I*_K_	−26	+ 6	*τ*_*n*_(*V*)	-	-	-
*I*_A_	−27(−41)	+ 13.2	5	−82(−95)	−6.5(−9.2)	10
*I*_T_	−54	+ 3	-	−74	−3.75	15
*I*_HVA_	−25	+ 8	4	-	-	-

**Table 2:** Parameter values of the full system (1) that do not change during runup while exhibiting tonic firing.

Par.	Value		Par.	Value		Par.	Value	
*C*	1.50148	*μ*F.cm^−2^	*g*_Na_	3.4	*μ*S.cm^−2^	*E*_Na_	+ 55	mV
*A*	322	ms.mV	*g*_K_	20.25	*μ*S.cm^−2^	*E*_K_	−80	mV
*y*_0_	0.1	ms	*g*_L_	0.07407	*μ*S.cm^−2^	*E*_L_	−38	mV
*V*_*c*_	-74	mV	*g*_A_	12.2	*μ*S.cm^−2^	*E*_K_	−80	mV
*w*	46	mV	*g*_T_	0.45045	*μ*S.cm^−2^	*E*_Ca_	+ 22	mV
*k*_*Ca*_	0.45	*μ*M	*g*_K(Ca)_	1.0	*μ*S.cm^−2^	*E*_K_	−80	mV
*α*	0.018	*μ*M.cm^2^.*μ*A^−1^.ms^−1^	*g*_HVA_	0.08	*μ*S.cm^−2^	*E*_Ca_	+ 22	mV
*k*	0.1	ms^−1^	*ε*	0.015	-	-	-	-

**Fig 1:**
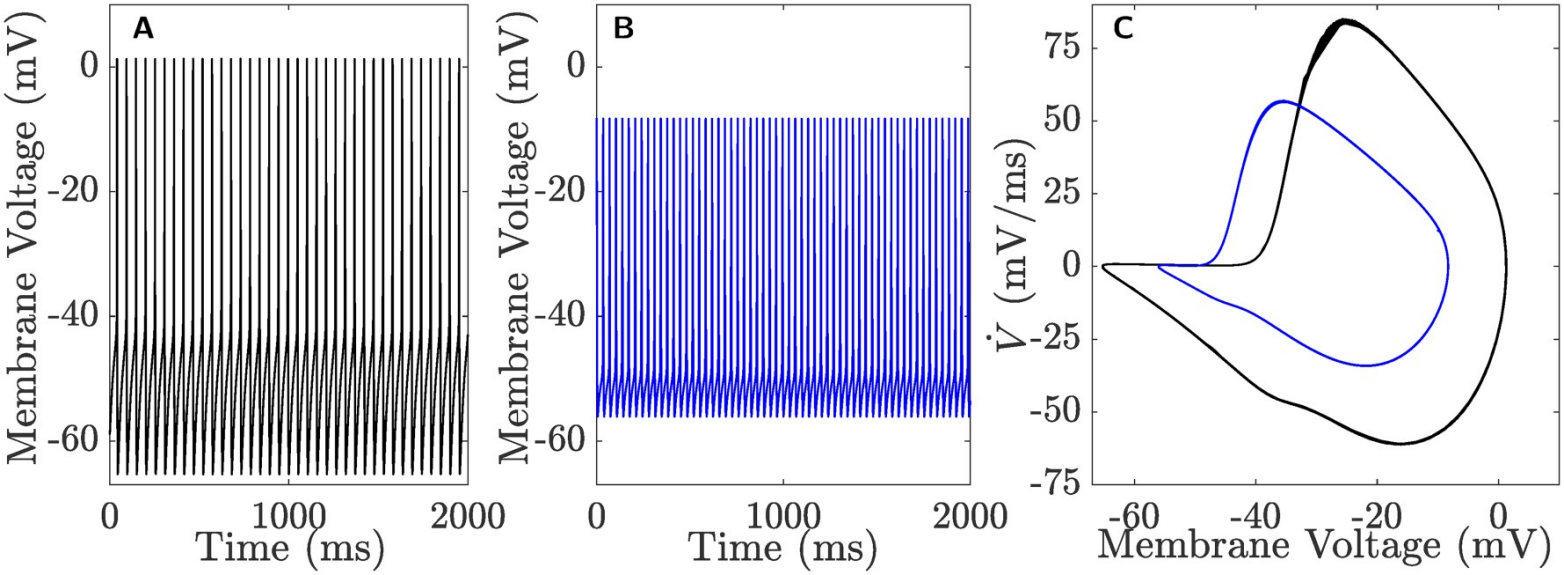
Time courses of the membrane voltage (*V*) during **A**) pre- and **B**) post-runup when the full system (1) is tonically firing. **C**) The AP-cycles of the same time courses in **A** and **B** plotted in the $\left(V,\dot{V}\right)$-plane. Black: pre-runup; blue: post-runup.

Time-scale separation of the full system (1) can be performed in a manner similar to that outlined in [17] (see Fig 1 in that paper). This is done by first plotting the time courses of the derivatives of its state variables and then classifying them as fast/slow variables according to how much variation they exhibit in their time courses: the larger the variation the faster the variable. Based on this approach, the three variables *h*_A_, *h*_T_ and *Ca* have been identified as being the slowest variables in this slow-fast system (since they all exhibit the smallest variations with the same order of magnitude). In this study, we treat *h*_A_ and *Ca* as parameters when conducting slow-fast analysis. For a full list of definitions of concepts adopted from the field of dynamical systems used in this study, please see S1 Text.

### Software and numerical methods

Numerical simulations are performed using the freeware XPPAUT (developed by Bard Ermentrout and available online at http://www.math.pitt.edu/~bard/xpp/xpp.html), while bifurcation analysis is performed using the software package Auto [46, 47] for computing bifurcation diagrams and latency profiles. To ease visualization, the L_2_-norm of the full system (1) is used in Auto for plotting equilibria, defined by ${\left(\sum_{i=1}^{8}x_{i}^{2}\right)}^{1/2}$, and periodic solutions of period *T*, defined by ${\left(\int_{t=0}^{T}x_{i}dt/T\right)}^{1/2}$, where *x*_*i*_’s are the state variables of the full system (1). We adapt a two-point boundary value problem (2PBVP) technique to compute the profiles and stable manifolds as described in [17]. The codes for regenerating the figures are available online [48].

## Results

### Type I excitability, latency and switching during runup

It was shown in [13, 16, 17] that the revised Hodgkin–Huxley model, comprised of Na^+^, K^+^, leak, A-type K^+^ and T-type Ca^2+^ currents, was able to produce the four key features of CSCs, including, type I excitability, the non-monotonic first-spike latency, runup and switching in responsiveness. Indeed, using 2PBVP, the continuation method in Auto and slow-fast analysis, the dynamics underpinning these activities were elucidated. It is thus important to verify if the expanded full system (1) is able to maintain the properties of the revised model in [13, 16, 17] and reproduce these four features of CSCs.

**Fig 2:**
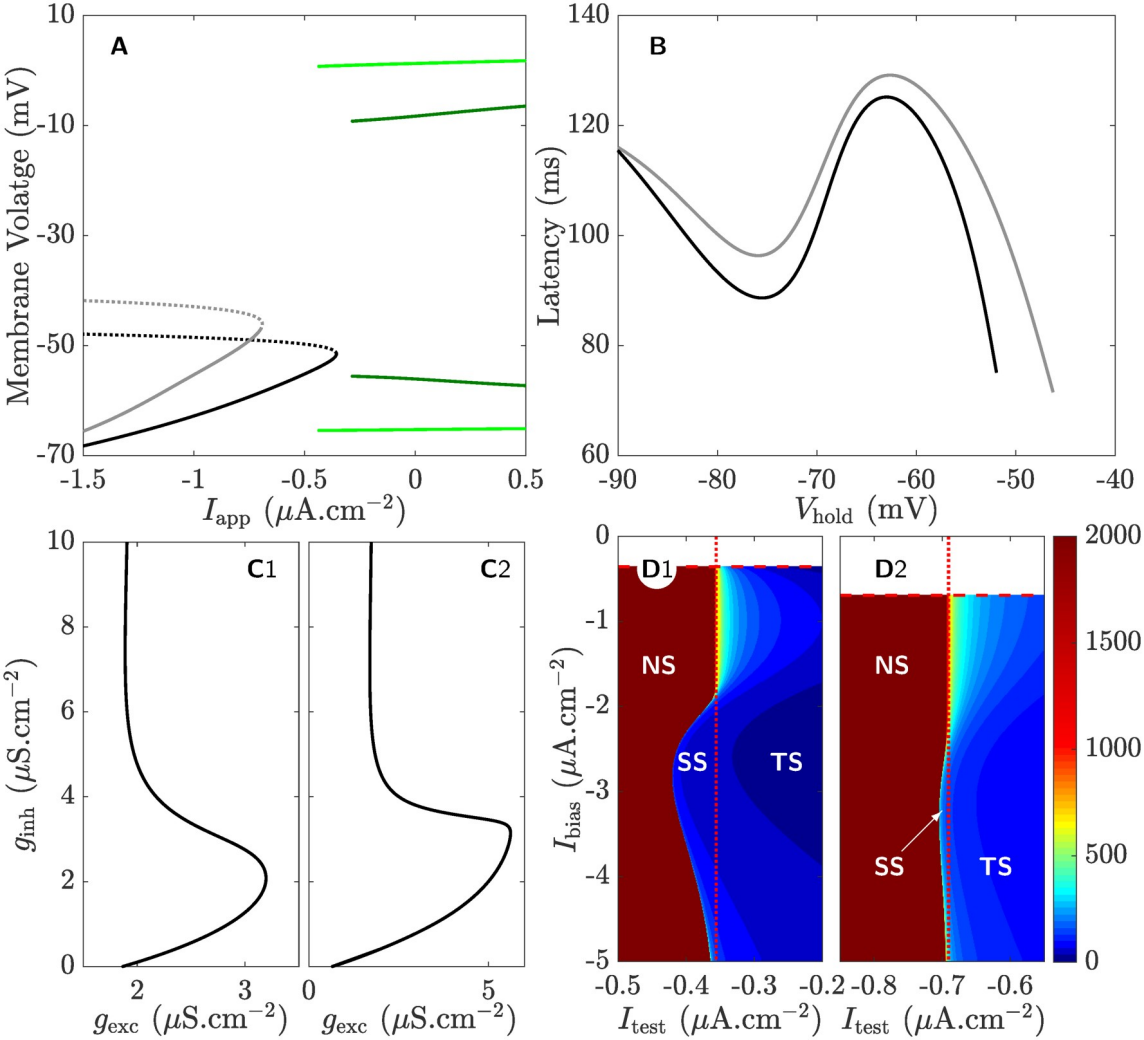
Review of the main four features of the full system (1): **A**) Type I excitability with a SNIC bifurcation during pre- (faded lines) and post-runup (dark lines). Solid (dashed) black/gray lines correspond to branches of stable/unstable equilibria, respectively; solid green lines correspond to envelopes of stable limit cycles. **B**) Non-monotonic first-spike latency with respect to the holding potential (*V*_hold_) during pre- (gray) and post-runup (back). **C**) The boundary between responsive (to the right) and non-responsive (to the left) regimes when a pair of inhibitory and excitatory pre-synaptic inputs of various magnitudes are applied during pre- (**C**1) and post-runup (**C**2). **D**) color-maps of the no-spiking (**NS**), single spiking (**SS**) and tonically spiking (**TS**) regimes during pre- (**D**1) and post-runup (**D**2), color-coded based on the duration of the first-spike latency calibrated according to the color-bar to the right. The system generates a single spike for *I*_test_ < *I*_SNIC_ when the stable manifolds of the saddle and saddle-node do not coincide and the initial condition lies between them. Time series simulations of these properties are available in [13, 16, 17].

With the inclusion of the two additional ionic currents, *I*_K(Ca)_ and *I*_HVA_, to the revised model, it is necessary to check if the resulting full system (1) is still able to capture the AP-cycle in the $\left(V,\dot{V}\right)$-plane and preserve the key dynamic features of CSCs. To do so, we first plot in Fig 1, the time series of the full system (1) describing CSCs as well as its AP-cycles in the $\left(V,\dot{V}\right)$-plane during both pre- (black) and post-runup (blue); the resulting simulations show that the AP-cycle look almost identical in characteristics to those produced experimentally [13]. To demonstrate that the model can capture all the other excitability features of CSCs, we plot in Fig 2:

1. The bifurcation diagram of the full system (1) with respect to the applied current (panel **A**) during both pre- (faded lines) and post-runup (dark lines). As demonstrated, the full system (1) exhibits type I excitability with a SNIC bifurcation in both cases.
2. The first-spike latency with respect to holding potential (panel **B**) during pre- (gray) and post-runup (black). The profile in both cases appears non-monotonic and identical to those seen previously [16].
3. The boundary between responsive (to the right) and non-responsive (to the left) regimes when the magnitudes of pre-synaptic inhibition *g*_inh_ and excitation *g*_exc_ are varied during pre- (panel **C**1) and post-runup (panel **C**2). As expected, switching in responsiveness occurs in both cases when *g*_inh_ is increased while *g*_exc_ is kept fixed (compare to [17]).
4. The boundary between no-spiking, single spiking and tonic spiking regimes in the *I*_bias_, *I*_test_-plane during pre- (panel **D**1) and post-runup (panel **D**2), where *I*_bias_ and *I*_test_ define the step current applied. In both cases, the tonic spiking regime (labeled **TS**) lies to the right of the stable manifold of the SNIC, while the single spiking regime (labeled **SS**) is limited, lying between the stable manifolds of the SNIC and saddle fixed point when they do not coincide [17]. These spiking regimes are color-coded according to the length of the first-spike latency (see color-bars), indicating that first-spike latencies become infinite (dark red) in the no-spiking regime (labeled **NS**) to the left of the spiking regimes **SS** and **TS**.

The above-listed results thus demonstrate that the full system (1) preserves all the dynamic properties of CSCs previously detected in the revised model developed in [13].

### Two modes of bursting in CSCs

#### Sqaure-wave bursting in the presence of low 4-AP

**Fig 3:**
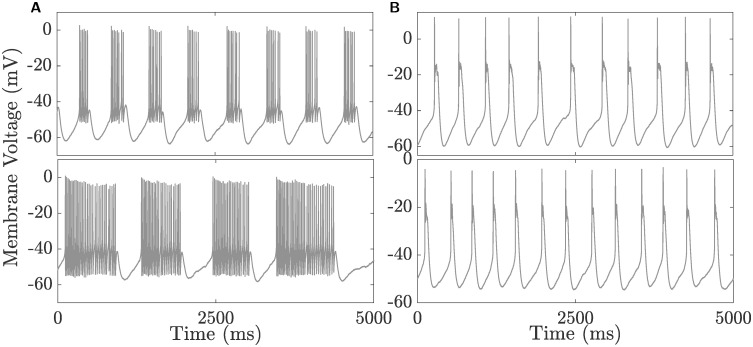
Two types of bursting recorded in CSCs. **A**) Square-wave bursting recorded in two CSCs bathed in 2 mM 4-AP, exhibiting short (top) and long (bottom) active phases. **B**) Pseudo-plateau bursting recorded in two CSCs bathed in 2 mM 4-AP and 300 *μ*M Cd^2+^, both exhibiting similar profiles.

Applying low doses of the pharmacological agent 4-AP, generally known to partially block *I*_A_ and *I*_K_, turns CSCs into bursting neurons with burst recordings characterized by a large first spike and non-uniform active phases with varying number of spikes. Fig 3**A** displays two such recordings obtained from two different cells bathed in 2 mM 4-AP with significant differences in the duration of their active phases and the number of spikes associated with these phases. The burst pattern in both recordings appear to suggest that these neurons are square-wave bursters, a claim that will be validated later on in the following sections.

#### Pseudo-plateau bursting in the presence of Cd^2+^ and low 4-AP

Another interesting feature about CSCs is their ability to exhibit another mode of bursting when cells are bathed not only in low dose (2 mM) of 4-AP, but also in 300 *μ*M Cd^2+^ (see the two recordings from two different cells in Fig 3**B**). These bursts are characterized by having active phases comprised of a spiking segment, followed by a plateau one; the spikes in the spiking segment are damped (i.e., decrease in amplitude) until they eventually disappear into the plateau segment. As we shall see later, this type of activity is consistent with pseudo-plateau bursting.

### Square-wave bursting in the CSC model

With the inclusion of the new ionic currents *I*_K(Ca)_ and *I*_HVA_ into the full system (1) and partially reducing the maximum conductances of *I*_A_ and *I*_K_ to different levels show that the model is unable to evoke any kind of bursting similar to those displayed in Fig 3**A** (results not shown). In fact, this outcome still persists even with the inclusion of other ionic currents known to be expressed on CSCs and to enhance bursting activity (results not shown).

**Fig 4:**
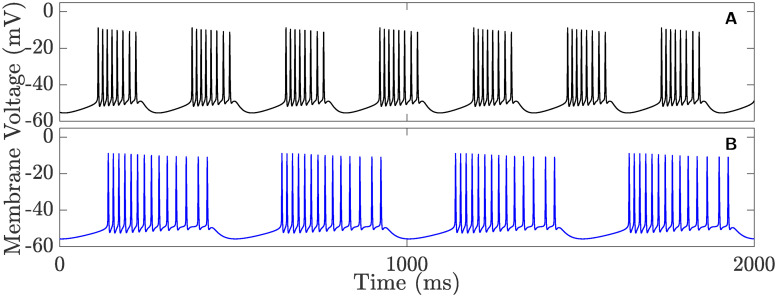
Two time courses of the membrane voltage (*V*) produced by the full system (1) during post-runup. Both exhibit bursting when **A**) (*g*_HVA_, *g*_A_) = (0.26, 6.0) *μ*S.cm^−2^, and B) (*g*_HVA_, *g*_A_) = (0.235, 12.2) *μ*S.cm^−2^.

It is typically assumed that any kind of bursting activity in cells is associated with oscillations in cytosolic Ca^2+^ concentration ([Ca^2+^]_*i*_) [49–51]. We therefore hypothesize that the application of certain low doses of 4-AP is inducing an increase in [Ca^2+^]_*i*_. To test this hypothesis, the maximum conductance of *I*_HVA_ (*g*_HVA_) has to be increased to see if this can cause the system to switch activity from tonic firing to square-wave bursting. Our results reveal that it is possible to induce this switching by targeting *g*_HVA_. Fig 4 shows two examples of such bursting activities during pre-runup produced by the full system (1) when *g*_HVA_ is increased and *g*_A_ is decreased from their default values listed in Table 2 to (*g*_HVA_, *g*_A_) = (0.26, 6.0) *μ*S.cm^−2^ (panel **A**) and (*g*_HVA_, *g*_A_) = (0.235, 12.2) *μ*S.cm^−2^ (panel **B**). Similar outcomes are also obtained when the maximum conductance of *I*_K_ is also slightly decreased in combination with altering the other two (results not shown). Although 4-AP is known to partially block the voltage-gated A-type and delayed-rectifier K^+^ currents *I*_A_ and *I*_K_, respectively, its ability to potentiate *I*_HVA_ appears to be unexpected. Evidence of such a potentiating effect of 4-AP, however, has been previously documented [52–54]. Indeed, it was shown that 4-AP can potentiate some of the sub-types of HVA currents, including N-, P/Q- and L-types Ca^2+^ currents, by threefold [52].

Considering how 4-AP alters *g*_A_ and *g*_HVA_ at low doses and how these maximum conductances may fluctuate under different physiological conditions, we will vary in the next section these parameters in the full system (1) and explore how they influence the electrical properties of CSCs.

### Number of spikes in a square-wave burst is sensitive to changes in the maximum conductance of *I*_HVA_

As mentioned above, low doses of 4-AP appears to potentiate *I*_HVA_ in CSCs. Applying different doses of 4-AP to a heterogeneous population of these cells can thus lead to differences in the way they respond. To understand the underlying dynamics of such differences, we use the full system (1) to investigate the effect(s) of varying the maximum conductances of the ionic currents, particularly those partially blocked and/or potentiated by 4-AP, on electrical activities of CSCs including bursting. We begin first by targeting the maximum conductance of *I*_HVA_ (*g*_HVA_) in the full system (1) during post-runup to see how it effects the active phases of burst firing. Note that similar results can be also obtained during pre-runup (results not shown).

**Fig 5:**
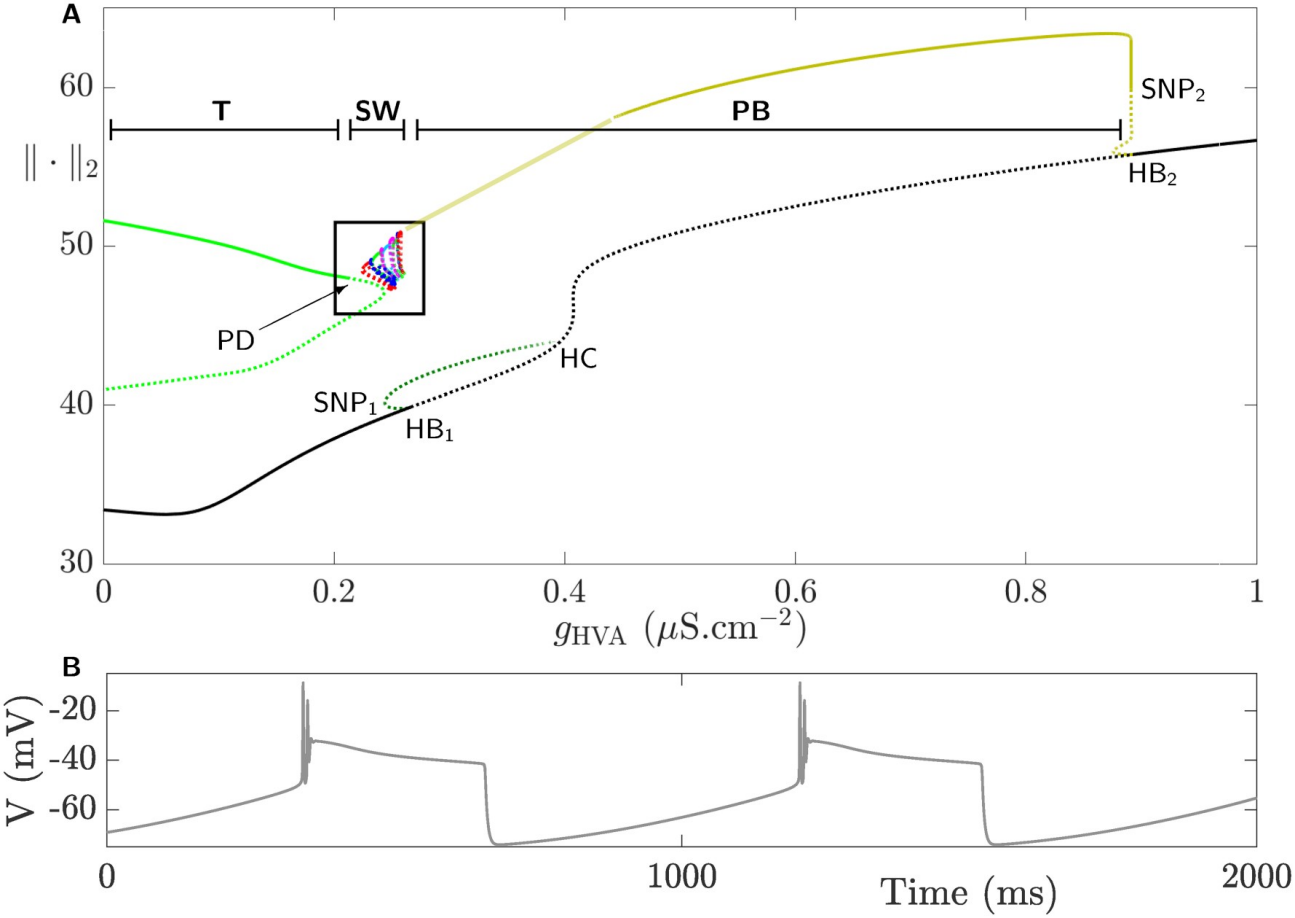
The effects of *g*_HVA_ on the dynamics of the full system (1). **A**) Bifurcation diagram of the full system (1) with respect to *g*_HVA_ using the L_2_-norm of the state variables. The branch of equilibria (black) undergoes two Hopf bifurcations, labeled HB_1_ and HB_2_. The equilibria are unstable (dotted) between HB_1_ and HB_2_ and stable (solid) otherwise. The envelope of unstable periodic orbits (dotted dark green) emanating from HB_1_ undergoes a saddle-node bifurcation of periodic orbits (SNP) and terminates at a homoclinic bifurcation, denoted HC. The envelope of periodic orbits (dark olive) generated from HB_2_ is unstable, but becomes stable at a saddle-node bifurcation of periodic orbits (SNP) and terminates at a homoclinic bifurcation very close to the right of HB_1_ (not shown). The light olive line is the continuation of the dark olive line obtained by numerical integration of the full system (1). Both correspond to the envelope of pseudo-plateau BPOs (labeled **PB**). The ⊃-shaped curve (light green) is an isola of POs corresponding to tonic firing (labeled **T**); it consists of two branches separated by a saddle-node bifurcation of periodic orbits (SNP_∞_). The upper branch is stable and becomes unstable at a period-doubling bifurcation (PD). The set of (colorful) curves to the right of PD within the box shows a family of more isolas of square-wave BPOs (labeled **SW**) (for better visualization, see the magnification of this box in Fig 6**A**). **B**) Time course of a pseudo-plateau BPO for *g*_HVA_ = 0.4 *μ*S.cm^−2^.

**Fig 6:**
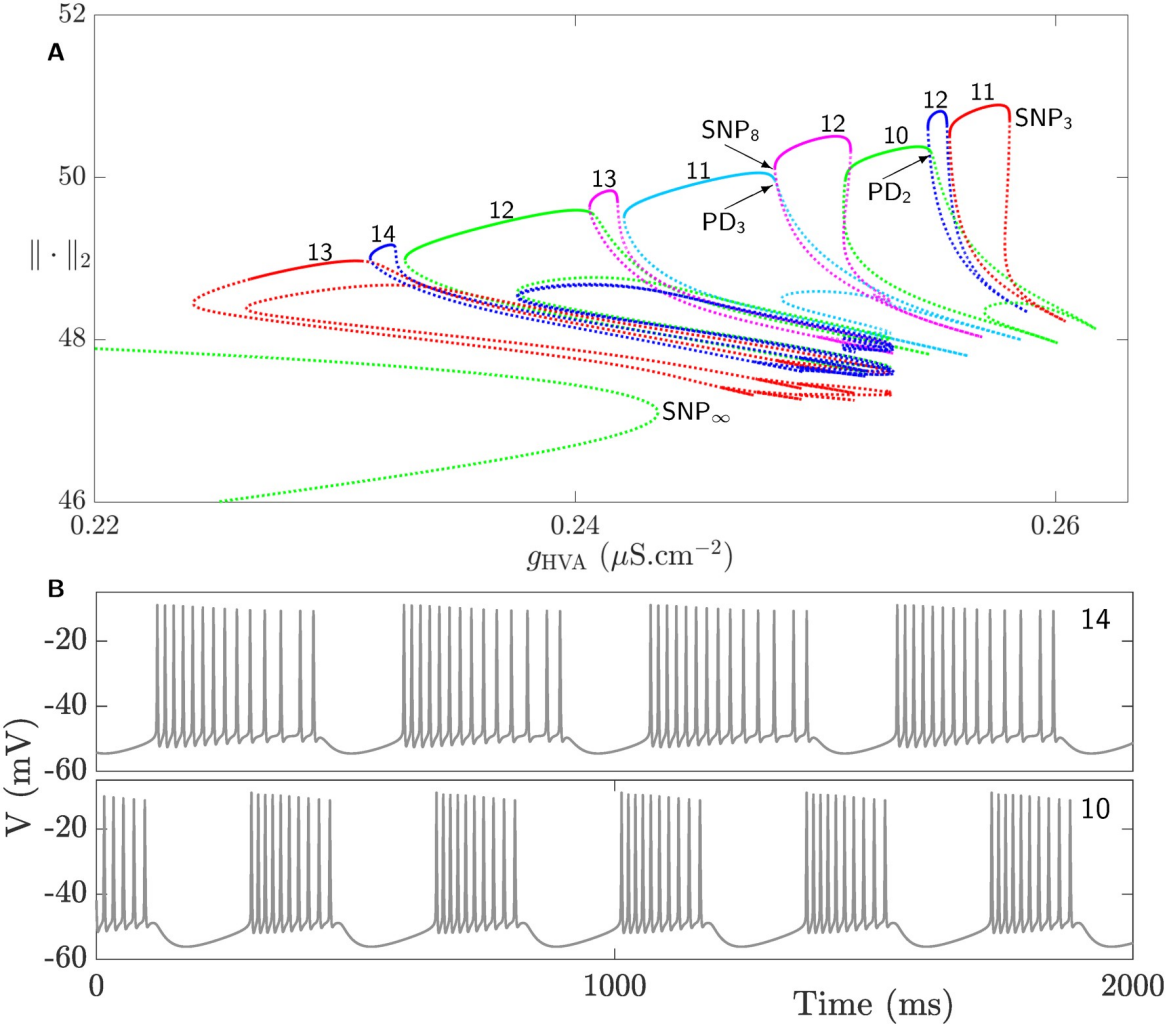
Square-wave bursting in the CSC model defined by the full system (1) when *g*_HVA_ is varied. **A**). Magnification of the set of isolas of square-wave BPOs shown within the box in Fig 5**A**. Each isola is an envelope of stable periodic orbits (solid) on the top that becomes unstable (dotted) either at a saddle-node bifurcation of periodic orbits (SNP_i_, i = 3, …) or period-doubling bifurcation (PD_j_, j = 1, …) to the right and left of each isola in a non-orchestrated manner (i.e., in a non-consistent pattern). The number of spikes of active phases of BPOs associated with these isolas is indicated with a number on top. **B**) Time courses of two square-wave BPOs when the number of spikes per burst is 14 (top) and 10 (bottom) obtained at g_HVA_ = 0.232 *μ*S.cm^−2^ and g_HVA_ = 0.253 *μ*S.cm^−2^, respectively.

Fig 5**A** displays the bifurcation diagram of system (1) with respect to *g*_HVA_, showing families of stable (unstable) equilibria and periodic orbits plotted in solid (dotted) black and colored lines, respectively. The branch of equilibria (black solid) is stable for small values of *g*_HVA_; the equilibria on this branch subsequently lose stability and regains it at two subcritical Hopf bifurcations, denoted HB_1_ and HB_2_, respectively. The unstable envelope of periodic orbits (dark green) emanating from HB_1_ terminates at a homoclinic bifurcation (HC) after undergoing a saddle-node bifurcation of periodic orbits (SNP_1_). The envelope of periodic orbits (olive) emanating from HB_2_ is also unstable but becomes stable at a saddle-node bifurcation of periodic orbits (SNP_2_). The continuation of this envelope of periodic orbits (represented with a faded olive line) is quite challenging but one can confirm, through numerical integration of the full system (1) as shown in Fig 5**B**, that it corresponds to stable pseudo-plateau BPOs (to be discussed later) and that it persists for the whole range of parameters up to the family of isolas of square-wave BPOs (inside the box), where it then disappears at a homoclinic bifurcation to the right of HB_1_. The envelope of BPOs in light green (the ⊃-shaped curve) for small *g*_HVA_ shows an isola with two branches: a stable upper branch which becomes unstable after undergoing a period-doubling bifurcation (PD), and an unstable lower branch merging with the unstable component of the upper branch at a saddle-node bifurcation of periodic orbits (SNP_∞_ shown more clearly in Fig 6**A** which magnifies the content of the box in Fig 5**A**). Close to PD, there are many isolas (highlighted by different colors within the box), each comprised of an envelope of stable and unstable periodic orbits. Because these isolas are not easily discernible, we have only computed a small number of these isolas; they accumulate on the upper branch of the ⊃-shaped isola.

When *g*_HVA_ is small, which is the case under control conditions for CSCs, the system is tonically firing as indicated by the stable branch of the ⊃-shaped isola. Increasing *g*_HVA_ pushes the system across the period-doubling bifurcation (PD) into a square-wave bursting regime defined by the set of isolas within the box. For larger *g*_HVA_-values, the family of isolas disappears and the system switches to a new pseudo-plateau bursting regime defined by the envelope of BPOs in olive. Finally, to the right of SNP_2_, the system no longer oscillates and instead reaches an elevated steady state representing the depolarization block.

To better visualize the family of isolas enclosed within the box in Fig 5**A**, it is magnified in Fig 6**A**. Each isola in this figure is computed by the continuation of a BPO. All the isolas possess a stable segment on top that is delimited by a saddle-node bifurcation of periodic orbits (SNP_i_, i = 3, …) and a period doubling bifurcation (PD_j_, j = 1, …) to the right and left in a non-orchestrated manner (i.e., with no specific pattern maintained between the isolas). Note that additional bifurcation points may also occur along each isola but only those bifurcations where the stability of the upper envelope is lost are the ones labeled (see Fig 6**A**). The stable envelopes of these isolas define square-wave BPOs whose time courses look similar to those shown in Fig 6**B**. The number of spikes during the active phases of these BPOs are specified by the numbers on top of each stable envelope in Fig 6**A**.

There are peculiar irregularities in the shape of these isolas along with the number of spikes associated with them when *g*_HVA_ is varied. Whereas in classic examples of isolas in which the number of spikes between two neighboring ones increases sequentially one at a time [38, 55], in this CSC model, not only the transition in the number of spikes between two adjacent isolas is not sequential, but also two isolas with overlapping bistable regimes can differ by more than one spike. The other significant difference between the isolas in Fig 6**A** and those observed in bifurcation diagrams of the same kind in previous studies is the transition from one isola to the next. In former studies [38, 55], it was found that two large primary isolas are separated by a secondary one possessing BPOs that exhibit two alternating numbers of spikes between two consecutive bursts that combine the number of spikes of the two adjacent primary isolas.

Furthermore, in the CSC model, the stable envelopes of the isolas in Fig 6**A** overlap, creating small intervals of bistability. For the same value of *g*_HVA_ in these intervals, the system can generate bursting activities with different number of spikes starting from different initial conditions. Using slow-fast analysis, we will later provide a conjecture for the underlying dynamics of this particular aspect of this system.

### Maximum conductance of *I*_K(Ca)_ determines the dynamics of square-wave bursting

It is known that K(Ca) channels, namely BK, IK and SK channels, regulate the duration of active phases during burst firing. The suppression (potentiation) of these channels can prolongs (shortens) the burst episodes, i.e., active phases. Moreover, the conductance of these channels are gated by [Ca^2+^]_*i*_, previously formulated mathematically as a strictly increasing fifth-order Hill function [56]. Variations in [Ca^2+^]_*i*_ can alter the maximum conductance of *I*_K(Ca)_ (*g*_K(Ca)_); therefore, it is imperative to provide a full bifurcation analysis of the full system (1) with respect to *g*_K(Ca)_ to determine how this affects the number of spikes in a BPO.

**Fig 7:**
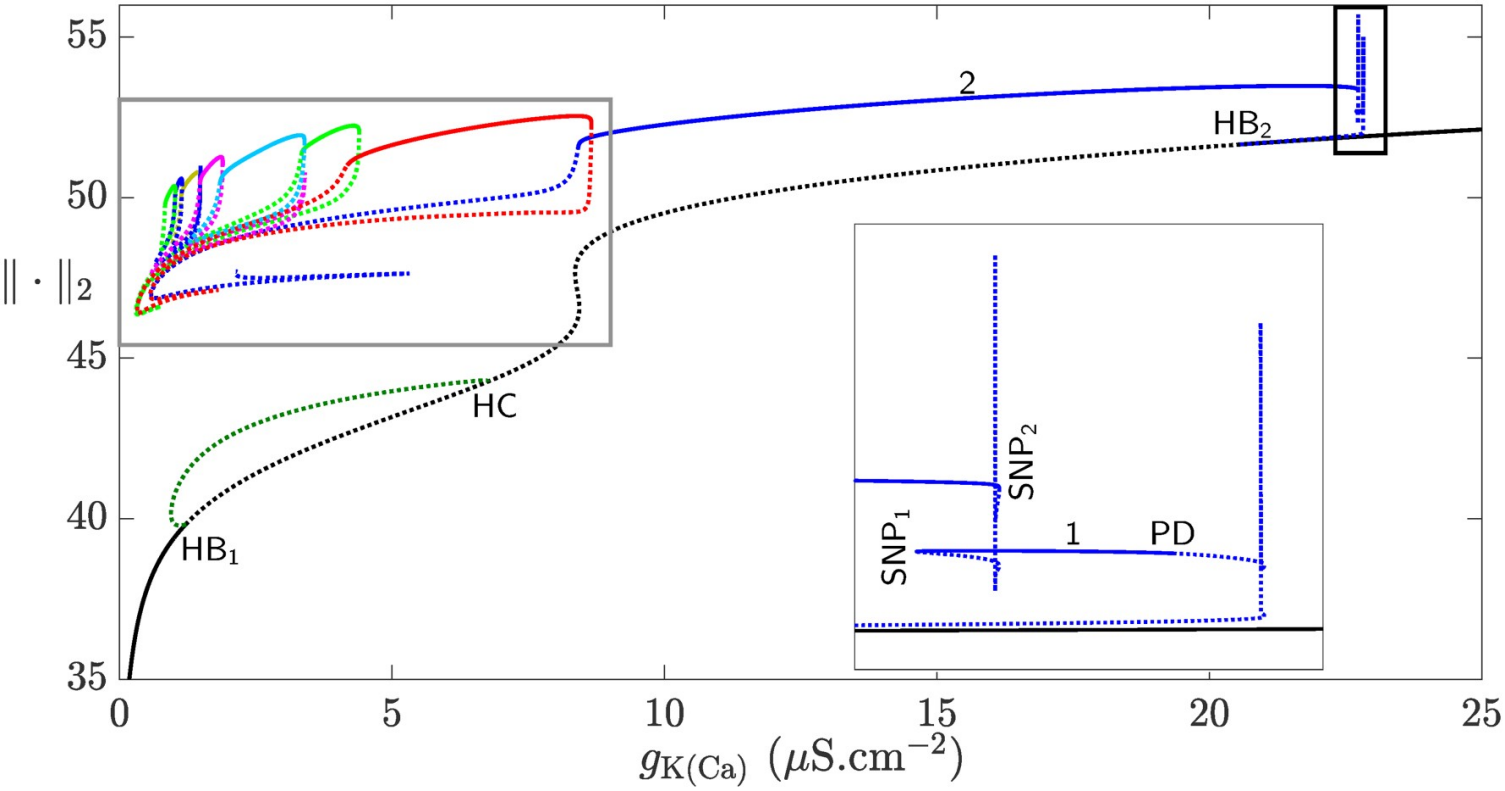
The effects of *g*_K(Ca)_ on the dynamics of the full system (1). Bifurcation diagram of the full system (1) with respect to *g*_K(Ca)_ using the L_2_-norm of the state variables. Two subcritical Hopf bifurcations HB_1_ and HB_2_ are present along the branch of equilibria (black). The equilibria are unstable (dotted) between HB_1_ and HB_2_ and stable otherwise (solid). The envelope of periodic orbits emanating from HB_1_ (olive) is unstable and terminates at a homoclinic bifurcation, denoted by HC. The family of periodic orbits emanating from HB_2_ is unstable. After a drastic change in the L_2_-norm of the state variables, the envelope becomes stable at a period-doubling bifurcation (PD). This envelope becomes unstable at a saddle-node bifurcation of periodic orbits (SNP_1_) before undergoing the spike-adding process, and then becomes stable again at another saddle-node bifurcation of periodic orbits (SNP_2_). The two instances of drastic changes in the L_2_-norm of the state variables inside the right black box are further magnified in the inset. The set of (colored) curves inside the left gray box shows isolas of square-wave BPOs with different number of spikes. These isolas are further magnified in Fig 8**A**.

Fig 7 shows the bifurcation diagram of the full system (1) with respect to *g*_K(Ca)_. The solid (dotted) curves are associated with the stable (unstable) branches of equilibria (black lines) and/or envelopes of periodic orbits (colored lines). For small values of *g*_K(Ca)_, the system has a single attracting equilibrium which becomes unstable at a subcritical Hopf bifurcation (HB_1_) for higher values of *g*_K(Ca)_. At HB_1_, the envelope of unstable periodic orbits (olive) emanating from it, undergoes a saddle-node bifurcation of periodic orbits before terminating at a homoclinic bifurcation, denoted by HC. The branch of equilibria regains its stability at another subcritical Hopf bifurcation HB_2_ at *g*_K(Ca)_ ≈ 20 *μ*S.cm^−2^, where an envelope of unstable periodic orbits (blue) emerges. This family of periodic orbits undergoes a spike-adding process, accompanied initially by sharp increases and decreases in the L_2_-norm of the state variables within the black box (see the inset for magnification of this box). It becomes stable at a period-doubling bifurcation (PD) and deforms into a single-spike BPO. Shortly after, this single-spike BPO changes into a two-spike BPO for lower *g*_K(Ca)_ values. The number of spikes in the bursting regime remains unchanged until the envelope of periodic orbits becomes unstable at a period-doubling bifurcation and a sequence of overlapping isolas of square-wave BPOs with different number of spikes emerges (gray boxed region to the left in Fig 7).

**Fig 8:**
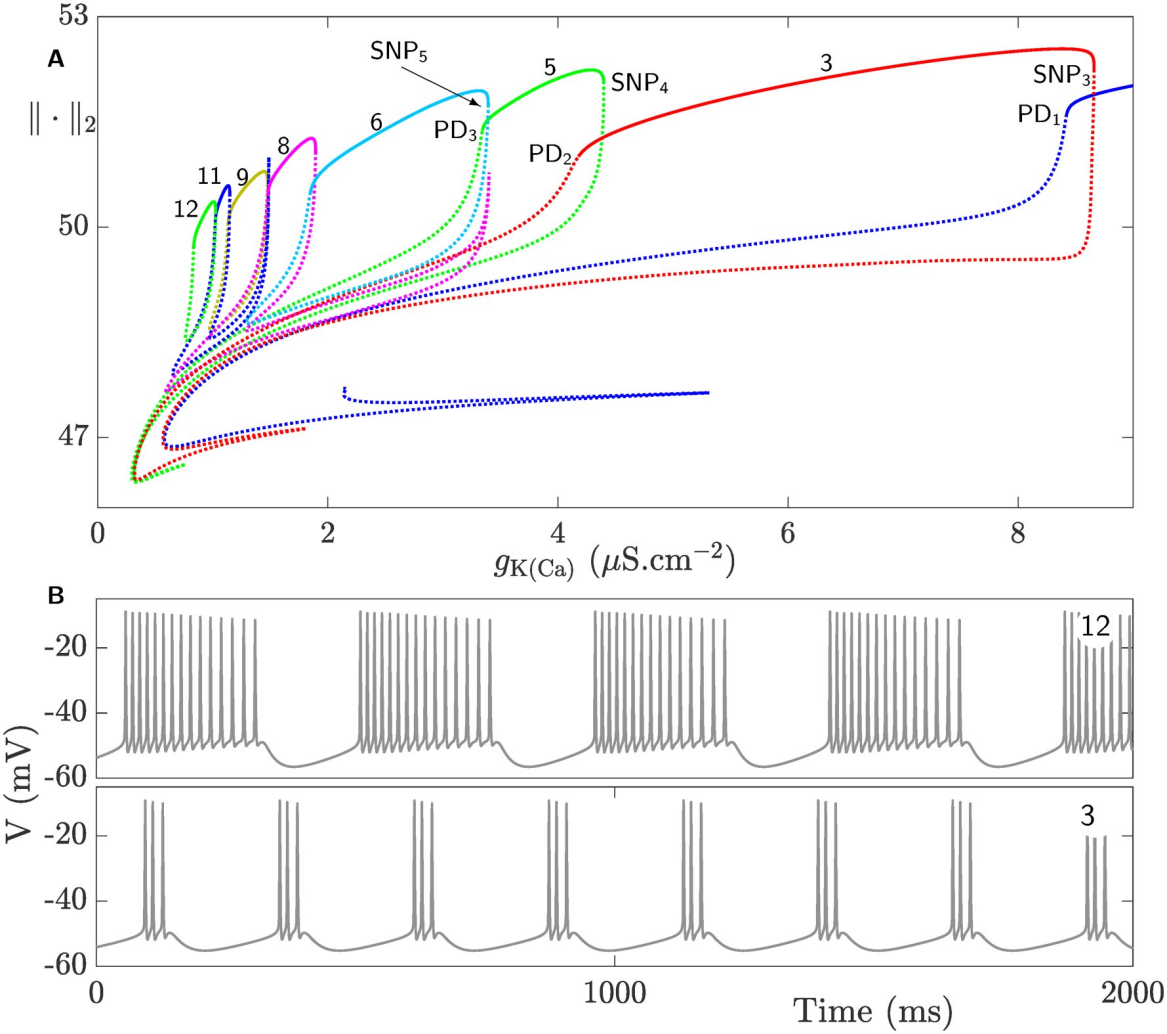
Square-wave bursting in the CSC model defined by the full system (1) when *g*_K(Ca)_ is varied. **A**) Magnification of the set of isolas of square-wave BPOs in the left black boxed region of Fig 7. The top segment of each isola is an envelope of stable periodic orbits (solid) that becomes unstable (dotted) at a saddle-node bifurcation of periodic orbits (SNP_i_, i = 3, …) from the right and period-doubling bifurcation (PD_j_, j = 1, …) from the left. The number of spikes for each isola is indicated by the number on top. **B**) Time courses of two square-wave BPOs when the number of spikes per burst is 12 (top) and 3 (bottom) obtained at *g*_K(Ca)_ = 0.9 *μ*S.cm^−2^ and *g*_HVA_ = 6 *μ*S.cm^−2^, respectively.

To better understand how the isolas in the left box of Fig 7 behave and overlap, they are further magnified in Fig 8**A**. The number on top of each isola in Fig 8**A** indicates the number of spikes of the square-wave BPOs associated with it (see Fig 8**B** for two examples of time courses of such BPOs). As in Fig 6**A**, the number of spikes does not increase in sequential manner between adjacent isolas and small regimes of bistability exist between them. Contrary to Fig 6**A**, however, the isolas in Fig 8**A** lose their stability in an orchestrated manner. More specifically, their stable segments are delimited by saddle-node of periodic orbits and period-doubling bifurcation to the right and left, respectively.

These results thus provide a clear evidence that K(Ca) maximum conductance is a key determinant of bursting activity of the CSC model. The irregularity exhibited by the isolas of BPOs associated with this parameter suggests that there are complex interactions between this conductance and HVA maximum conductance. To uncover these complex interactions, one needs to perform a two-parameter bifurcation analysis involving the maximum conductances of *I*_K(Ca)_ and *I*_HVA_.

### Characterizing square-wave bursting dynamics and spike adding in parameter space

We have shown that bursting dynamics of the full system (1) is governed by a family of isolas of square-wave BPOs whose number of spikes changes in a non-sequential manner between adjacent isolas. We have also established that there are regimes of bistability between two adjacent isolas delimited by a saddle-node bifurcation of periodic orbits or a period-doubling bifurcation. Here, we continue this bifurcation analysis in two-parameter space to determine the regions with the same number of spikes and their boundaries.

**Fig 9:**
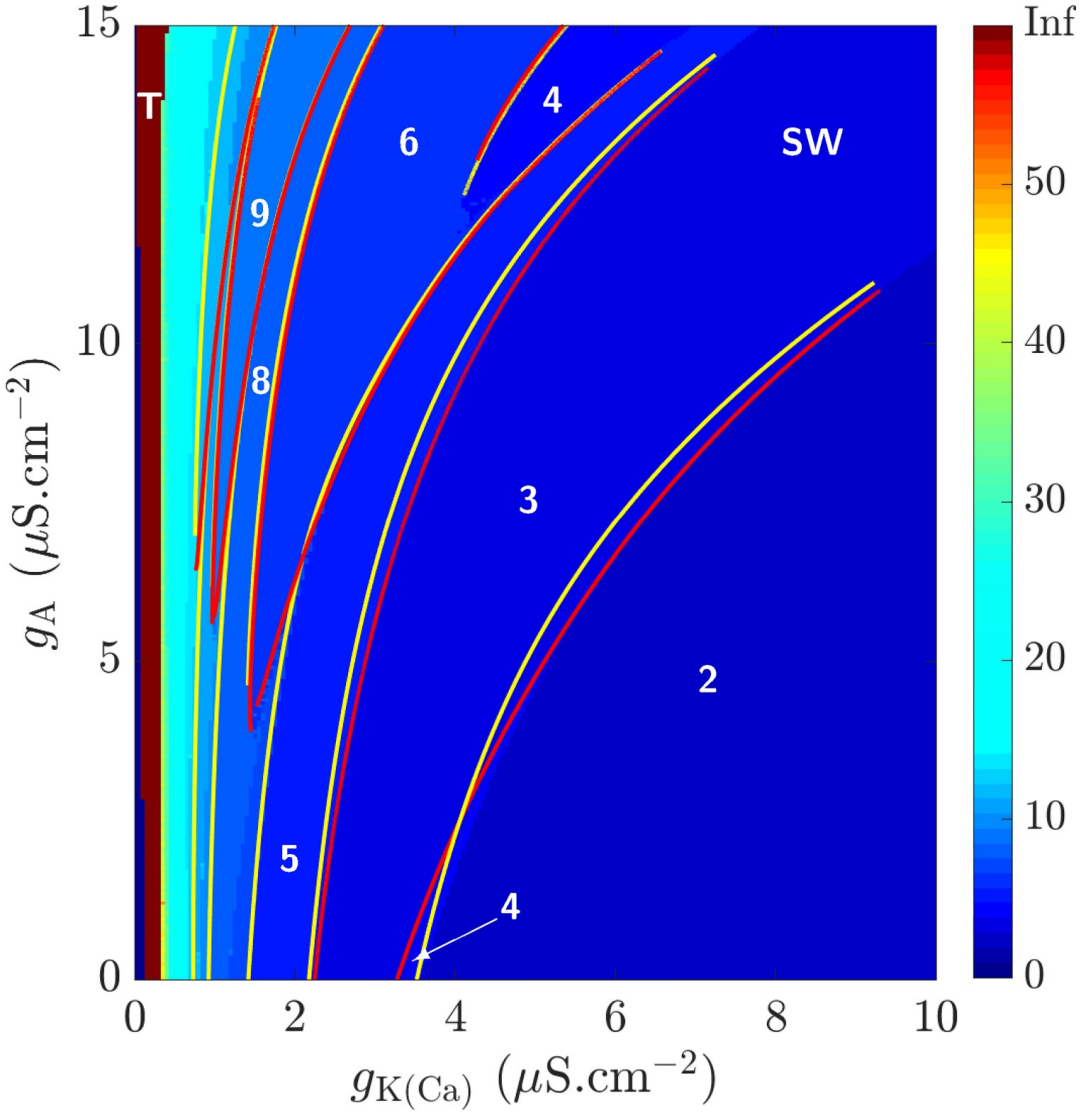
Color-map of the various regimes of behavior associated with the full system (1) in the (*g*_K(Ca)_, *g*_A_)-plane, when *g*_HVA_ = 0.249 *μ*S.cm^−2^. The square-wave bursting regime, labeled **SW**, is color-coded based on the number of spikes in its corresponding BPOs, calibrated according to the color-bar to the right. For further clarity, the number of spikes in various regions of **SW** are displayed. The number of spikes increases as *g*_K(Ca)_ decreases such that for small values of *g*_K(Ca)_, the system transitions to tonic firing (labeled **T**). The yellow and red curves are two-parameter continuation of the period-doubling and saddle-node bifurcations of periodic orbits detected in Fig 8**A**, respectively.

As mentioned previously, CSCs bathed in low doses of 4-AP alter their firing properties from tonic firing to bursting due to the partial blockage of *I*_A_ (and *I*_K_) and the potentiation of *I*_HVA_. However, this blockage and potentiation may vary between cells and are likely due to differences in the expression levels of these ionic channels in CSCs as well as the underlying dynamics of [Ca^2+^]_*i*_. Therefore, it is important to investigate how the number of spikes are organized in the (*g*_K(Ca)_, *g*_A_)-plane when *g*_HVA_ is kept fixed at 0.249 *μ*S.cm^−2^. This is done in the color-map of Fig 9, plotted in the square-wave bursting regime (labeled **SW**) and color-coded based on the number of spikes according to the color-bar to the right. As *g*_K(Ca)_ decreases, the number of spikes increases until it finally switches to tonic firing (labeled **T**) close to *g*_K(Ca)_ = 0 *μ*S.cm^−2^.

To first understand what underlies the irregularities in spike adding, it is imperative to define the boundaries of the different bursting regimes. This is done by further applying the two-parameter continuation method to plot in Fig 9 the curves of saddle-node bifurcations of periodic orbits (red) and the curves of period-doubling bifurcations (yellow), defining the boundaries between the different spiking regimes. As shown, these curves lie very tightly close together along the boundary. The narrow bands between the SNP curve (to the left) and PD curve (to the right) define bistable regimes of overlapping stable envelopes of two adjacent isolas. In the reverse case when the SNP curve is to the right of the PD curve, there are additional isolas that lie in between which produce bistable regimes of their own in a manner just described. When these curves cross each other and switch their position, they create regions with different number of spikes due, as stated before, to additional isolas in between (see for example region **4**). Note that the narrow burgundy band for very small *g*_K(Ca)_ on the left corresponds to the parameter regime (labeled **T**) where the model tonically fires. These results thus suggest that the presence of period doubling bifurcations is responsible for the non-sequential spike adding between adjacent isolas.

With this irregularity in spike adding, it would make more sense to talk about the global trend of this phenomenon rather than the increment change in the number of spikes. The number of spikes in the **SW** regime slightly increases when *g*_A_ is increased, in contrast to the significant increase in the number of spikes observed when *g*_K(Ca)_ is decreased. Therefore, the non-sequential order of spike adding between adjacent isolas makes the creation (termination) of a region more likely to happen along a variation in *g*_A_ compared to other parameters.

### Slow-fast analysis underlying square-wave bursting

In this section, we focus on the underlying dynamics of the full system (1) that give rise to square-wave bursting activities, taking advantage of the significant differences in time scales of the variables present in the model. To do so, we apply slow-fast analysis in its classical sense [26–28], treating the variables that evolve slowly as parameters and focusing on the dynamics of the so-called fast subsystem formed by the remaining variables. To do so, time-scale separation is performed on the full system (1) as described in [17] (see Methods Section for more details), where slow and fast variables are identified. Although three times scales exist in this model, we only consider here two time scales: slow and fast, when analyzing its underlying dynamics.

**Fig 10:**
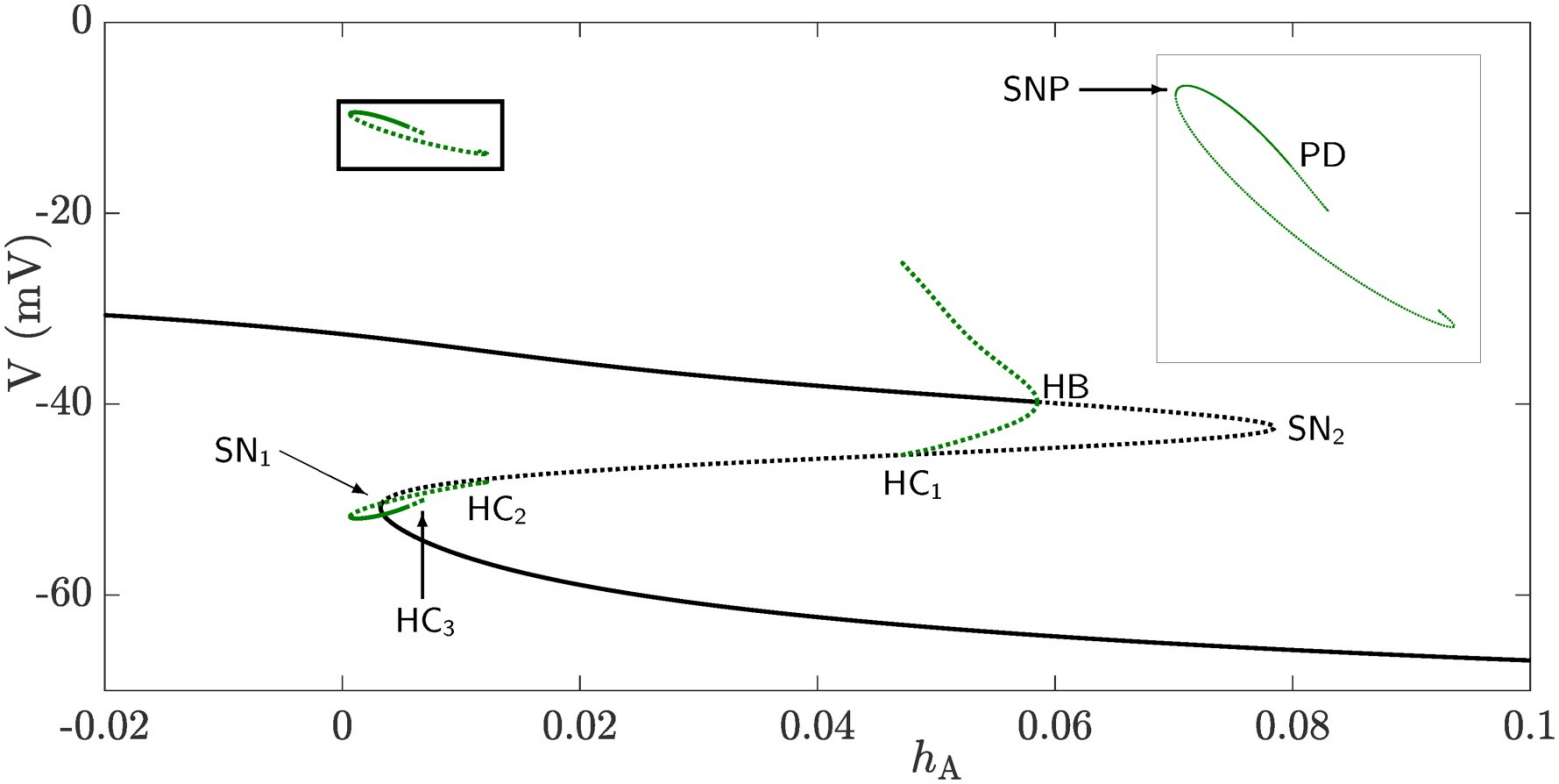
Bifurcation diagram of the fast subsystem, represented by the membrane voltage *V*, when *h*_A_ is varied as a parameter and *Ca* is kept fixed at a random but physiologically-relevant value of 0.2 *μ*M. The Z-shaped curve (black) is the branch of equilibria of the fast subsystem consisting of three branches separated by two saddle-node bifurcations, denoted SN_1_ and SN_2_. The solid and dotted black lines indicate stable and unstable branches of equilibria, respectively. The lower and middle branches are of stable and saddle type, respectively. The upper branch is stable for lower values of *h*_A_ and becomes unstable (of saddle type) at a subcritical Hopf bifurcation, denoted HB, where an envelope of unstable periodic orbits emanates and terminates at a homoclinic bifurcation (HC_1_). An isolated envelope of periodic orbits comprised of an upper and a lower ⊂-shaped loop exists near *h*_A_-values of SN_1_. The isolated envelope has an inner envelope of unstable periodic orbits that terminates at a homoclinic bifurcation (HC_2_) to the right, but undergoes a saddle-node bifurcation of periodic orbits (SNP) to the left, forming a stable outer envelope. As shown in the inset, this envelope becomes unstable at a period doubling bifurcation (PD) and terminates to the right at another homoclinic bifurcation, denoted HC_3_.

As indicated in the Methods Section, the variables *h*_A_ and *Ca* evolve much more slowly in time than the remaining set of variables; this means that they can be treated as parameters in the fast subsystem. Fig 10 shows the bifurcation diagram of the fast subsystem, represented by *V*, with respect to the slow variable *h*_A_ when *Ca* = 0.25 *μ*M. The solid (dashed) lines refer to stable (unstable) families of equilibria and periodic orbits. The family of equilibria forms a Z-shaped curve with three branches separated by two saddle-node bifurcations SN_1_ and SN_2_. The lower branch is stable, but becomes unstable at SN_1_. After passing through SN_2_, the branch of equilibria undergoes a subcritical Hopf bifurcation (HB) and becomes stable again. The envelope of periodic orbits emanating from HB is unstable and terminates at a homoclinic bifurcation, labeled HC_1_. There is also an isolated envelope of periodic orbits (magnified in the inset) close to the left knee of the Z-shaped curve (SN_1_) consisting of an upper and lower ⊂-shaped loops corresponding to the maximum and minimum of the limit cycles associated with this isolated branch, respectively. These two ⊂-shaped loops terminate at two homoclinic bifurcations at their two ends; these homoclinic bifurcations are labeled HC_2_ (to the right) and HC_3_ (to the left). The outer branches of the the two ⊂-shaped loops are stable, but become unstable to the right at a period-doubling bifurcation (PD) prior to HC_3_, and to the left at a saddle-node bifurcation of periodic orbits, denoted (SNP), to form the inner branches of the two ⊂-shaped loops.

**Fig 11:**
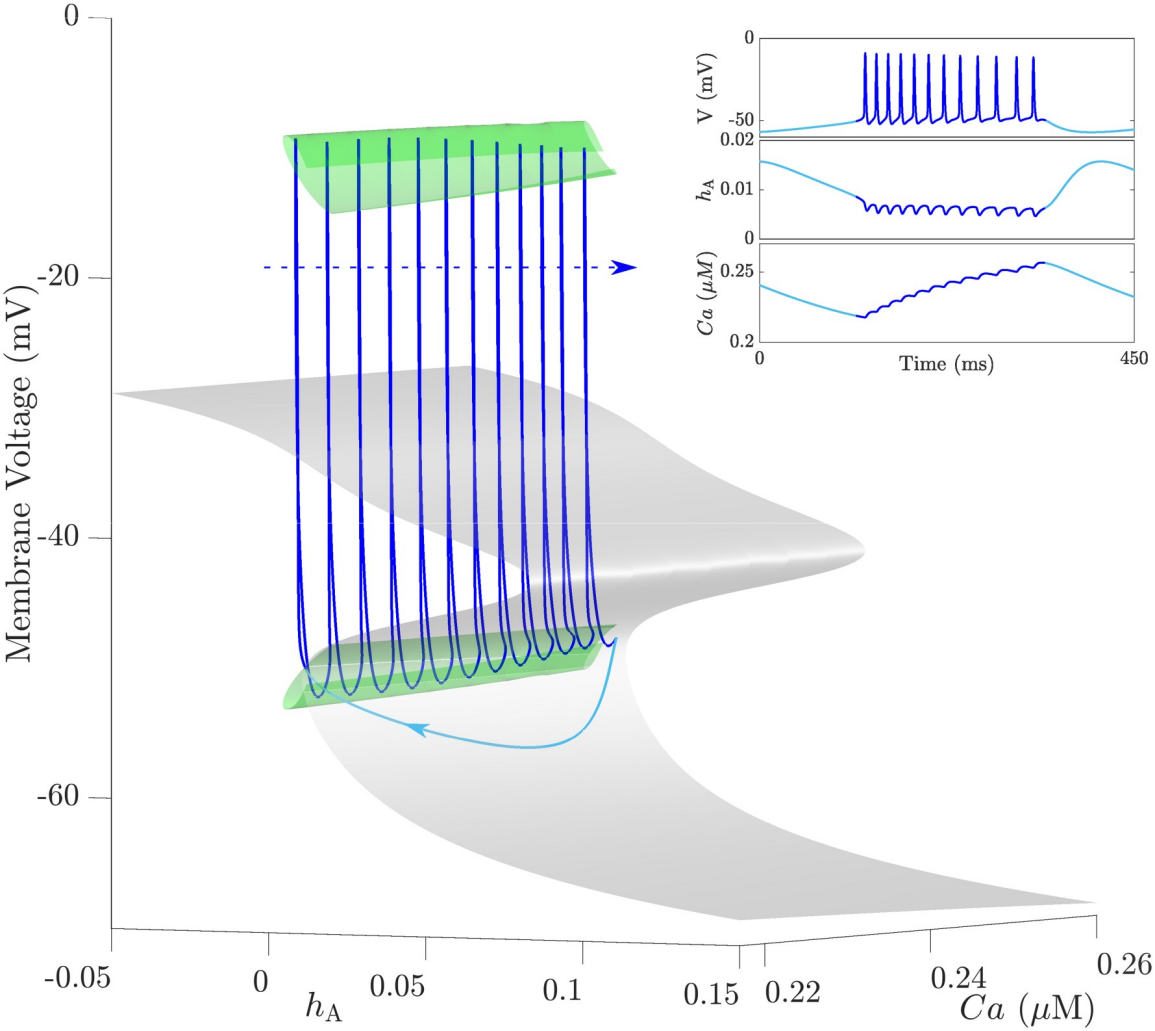
A three-dimensional representation of the bifurcation diagram shown in Fig 10 for a whole range of [Ca^2+^]_*i*_ (*Ca*). The gray surface is the critical manifold with three sheets of equilibria separated by two folds, whereas the green surface is a family of isolated envelopes of periodic orbits (as suggested by Fig 10). The lower/middle sheets of the critical manifold are attracting/repelling, respectively, while the upper sheet is attracting to the left of a curve of Hopf bifurcations (not shown), and repelling to the right. The sheets of unstable periodic orbits emanating from the curve of Hopf bifurcations are not shown. A square-wave bursting trajectory (blue) is superimposed on the bifurcation diagram. The arrows indicate the direction of flow and dark and light shades of blue discern between active and silent phases of the BPO, respectively. The inset shows the time courses of *V*, *h*_A_ and *Ca* corresponding to the square-wave BPO shown in the main panel.

To understand how the two slow variables *h*_A_ and *Ca* interact to produce bursting, we further compute a family of these bifurcation diagrams, similar to the one shown in Fig 10 for different values of [Ca^2+^]_*i*_. The resulting bifurcation diagram in the (*h*_A_, *Ca*, *V*)-space (shown in Fig 11) is a three dimensional structure that consists of two surfaces, one of which is the surface of equilibria called the critical manifold (gray) and another representing the family of isolated envelopes of periodic orbits (green). Superimposing a BPO (blue) on this bifurcation structure shows how these two surfaces govern burst dynamics (for the time courses of *V*, *h*_A_ and *Ca* of this BPO, see inset). As shown, the solution trajectory initially drops down to the lower stable sheet of the critical manifold and follows it up to the fold defined by SN_1_ (see Fig 10). As soon as the trajectory crosses the fold, it jumps up towards the family of stable periodic orbits created by the family of isolas and starts oscillating, causing an increase in [Ca^2+^]_*i*_. Because the stable sheet formed by the isolated envelopes becomes narrower for larger [Ca^2+^]_*i*_, the trajectory eventually crosses both the PD and HC_3_ bifurcations and returns back to the lower stable sheet of the critical manifold to repeat the cycle. The direction of the solution trajectory is either towards increasing *Ca* during spiking, or towards decreasing *Ca* during the silent phase. Since spiking in the active phase of the BPO follows closely the family of isolated envelopes until the homoclinic while the silent phase follows the bottom attracting sheet of the critical manifold until the fold, this type of bursting resembles very closely that associated with square-wave bursting, hence the terminology.

### Underlying dynamics of bursting in CSCs

**Fig 12:**
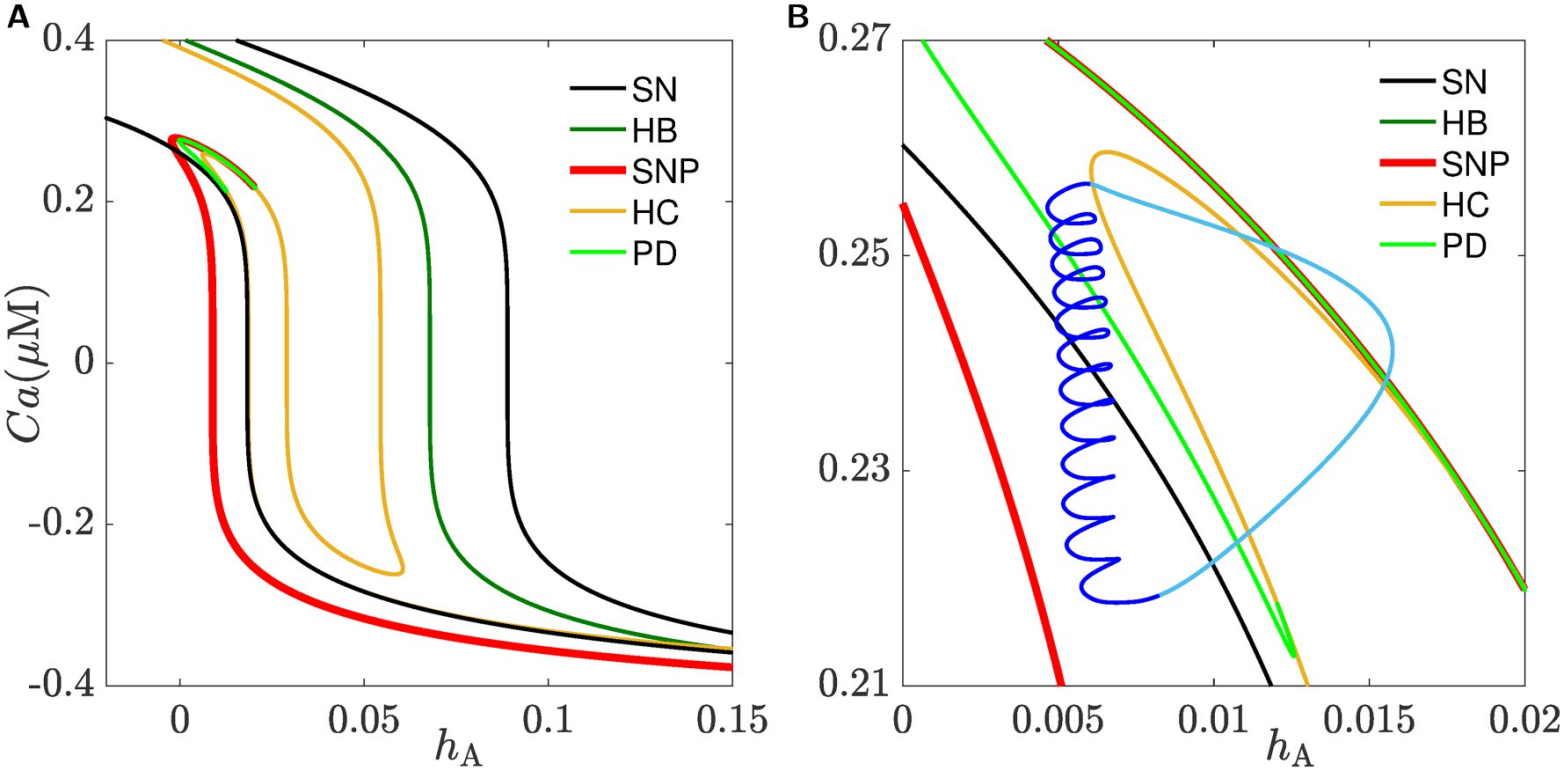
Two-parameter continuation of the bifurcation points in Fig 10. **A**) Two-parameter continuation of the bifurcation points SN_1_, SN_2_, HB, SNP, HC_1_, HC_2_ and HC_3_ of the fast subsystem detected in Fig 10 plotted in the *h*_A_, *Ca*-plane. **B**) The burst cycle (blue) is superimposed on an enlargement of the figure in **A**; the arrows indicate the direction of flow and dark and light shades of blue distinguish between active and silent phases of the square-wave BPO, respectively. The curves produced by the bifurcation points are color-coded according to the legend in each panel.

Since Fig 11 does not provide a clear picture as to how a BPO is initiated and terminated, we need to adopt another approach that can provide insights into the organization of bifurcation points in the parameter space. This is done by applying a two-parameter continuation of the bifurcation points detected in Fig 10 over the (*h*_A_, *Ca*)-plane which produces the bifurcation curves shown in Fig 12**A**, color-coded according to the legend. As shown, the curves of saddle-node bifurcations (black), denoted SN, and Hopf bifurcations (green), denoted HB, are almost identical copies of one another. The curves of the other bifurcation points follow almost the same trend but they have a more intricate shapes. The curve of homoclinic bifurcations (orange), denoted HC, is folded three times in the middle, indicating the coexistence of three homoclinic bifurcations for a fixed *Ca*-value as is the case in Fig 11. The (thick) curve of saddle-node bifurcation of periodic orbits (red), denoted SNP, lies to the left most. It folds for high [Ca^2+^]_*i*_ and then terminates at a codimension-two Belyakov point [57]. The curve of period-doubling bifurcations (light green), denoted PD, lies between SNP and HC. It terminates from the right at a strong resonance 1:2 on SNP and from the left at a homoclinic flip bifurcation on HC [58, 59].

In panel **B** of Fig 12, an enlargement of panel **A** combined with a square-wave BPO (blue), obtained from one of the isolas in Fig 8**A** and superimposed on top of the bifurcation curves, are displayed; the color-coding of the bifurcation curves follows the same legend as in panel **A**. By examining these bifurcation curves, we see that when the BPO crosses the black SN curve, it lies in an area between SNP (red) and HC (orange) curves where a family of stable periodic orbits of the fast subsystem exists. This causes the trajectory to oscillate, producing the active phase of the burst. The termination of the active phase of the BPO, however, is not clear. Typically, a BPO switches to a silent phase because of either the loss of stability of the family of periodic orbits of the fast subsystem through a period-doubling bifurcation, or the disappearance of these periodic orbits at a homoclinic bifurcation. Indeed, our computations reveal that the BPO terminates within a *Ca*-interval delimited by HC (orange) and SNP (red) curves. We conjecture that the presence of the curve of PD and its subsequent cascade of period-doubling curves make the termination of oscillations less organized and spike adding between the isolas non-sequential. More concisely, we suggest that the existence of infinitely many saddle-type periodic orbits and the interaction of their stable and unstable manifolds play the major role in ending the active phases of the square-wave BPO.

### Pseudo-plateau and chaotic bursting in CSCs

**Fig 13:**
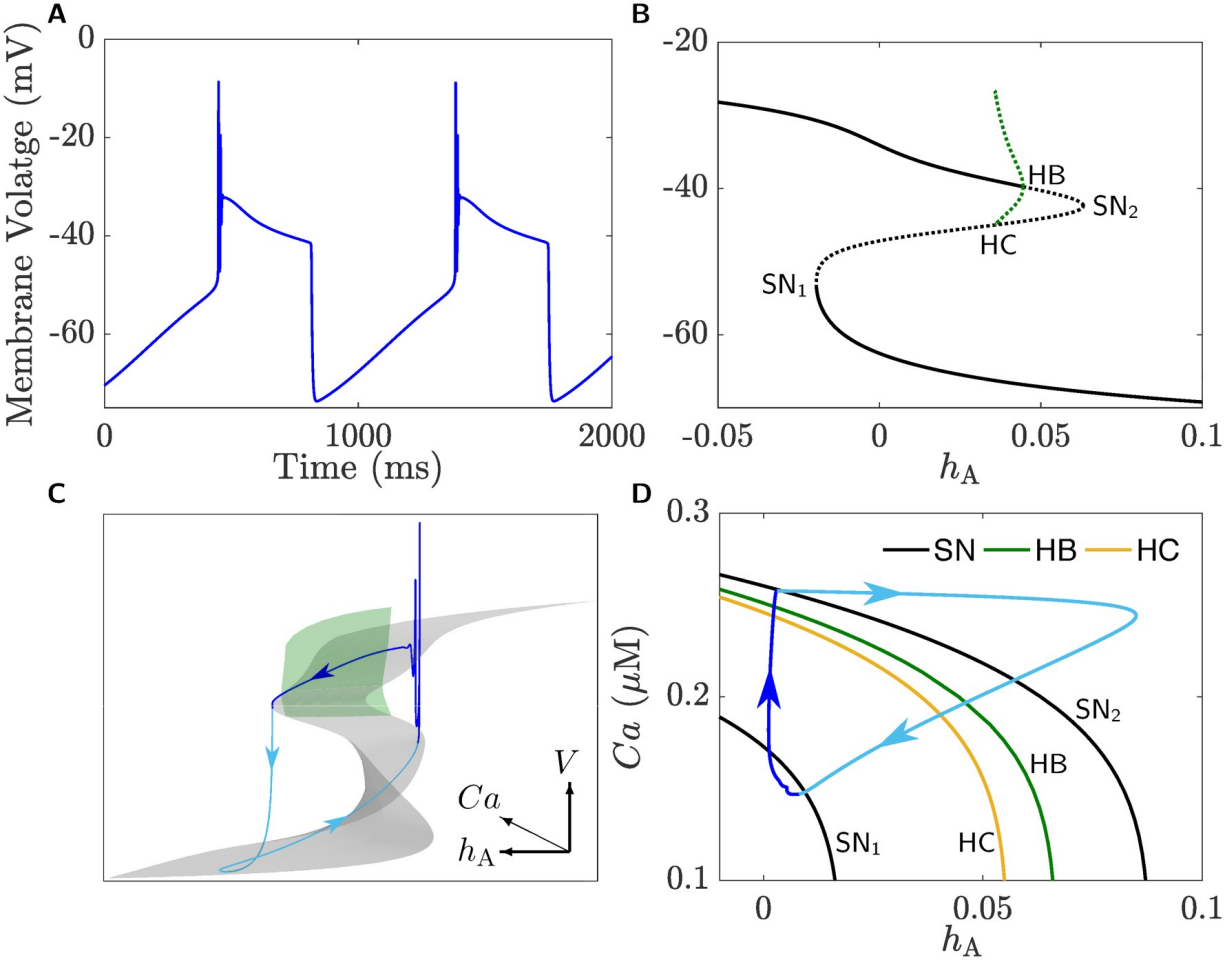
Pseudo-plateau bursting in the CSC model defined by the full system (1). **A**) Time course of the membrane voltage *V* when *g*_K_ = 12 *μ*S.cm^−2^, *g*_K(Ca)_ = 7 *μ*S.cm^−2^ and *g*_HVA_ = 0.22 *μ*S.cm^−2^. **B**) Bifurcation diagram of the fast subsystem, represented by the variable *V*, with respect to *h*_A_ using the same values of maximum conductances listed in **A**. As in Fig 10, the stable (unstable) branches of equilibria are plotted as solid (dotted) lines, while envelopes of unstable periodic orbits are plotted as green dotted lines. Notice the absence of the isola near SN_1_ seen in Fig 10. **C**) The BPO shown in panel **A** superimposed on the critical manifold of system (1) shown as a gray surface in the (*h*_A_, *Ca*, *V*)-space. The family of envelopes of unstable periodic orbits emanating from the curve of Hopf bifurcations lying on the upper sheet of the critical manifold is shown as a green surface. **D**) Two-parameter continuation of the bifurcation points SN_1_, SN_2_, HB and HC detected in panel **B** plotted in the (*h*_A_, *Ca*)-space along with one pseudo-plateau bursting cycle (blue); the flow direction is indicated with the arrows and active and silent phases are shaded dark and light blue, respectively. The bifurcation curves are color-coded according to the legend.

As already mentioned, the application of Cd^2+^ and a low dose of 4-AP to CSCs can give rise to a specific type of burst firing called pseudo-plateau bursting (see Fig 3**B**). Although such behavior can be also replicated by the full system (1) (see Fig 13**A**), the underlying mechanism for generating such activity is different from that seen in square-wave bursting (see Figs 10–12).

For the full system (1) to produce pseudo-plateau bursting (see Fig 13**A**), we have set *g*_K_ = 12 *μ*S.cm^−2^, *g*_HVA_ = 0.22 *μ*S.cm^−2^ and *g*_K(Ca)_ = 7 *μ*S.cm^−2^. The decrease in the maximum conductance of an already potentiated *I*_HVA_ by 4-AP is consistent with the expected effect of Cd^2+^, but the increase in the maximum conductance of *I*_K(Ca)_ seems to suggest that Cd^2+^ may have side effects (the decrease in *g*_K_ is expected in view of the fact that 4-AP partially blocks *I*_K_ which is assumed to be present). Indeed, such side effects have been documented in previous studies [60]. In other words, it seems that the effects of a low dose of 4-AP along with Cd^2+^ extend beyond what we know about them, and that a lower *g*_K_ is more needed for generating pseudo-plateau bursting compared to square-wave bursting.

By applying slow-fast analysis on the full system (1), we obtain in Fig 13**B** the bifurcation diagram of the fast subsystem, represented by the voltage variable *V*, with respect to *h*_A_ for a fixed *Ca* = 0.25 *μ*M. Comparing this bifurcation diagram to the one in Fig 10 reveals that they are almost identical except for the absence of the isolated envelope of periodic orbits close to SN_1_ in Fig 13**B**. By plotting a family of such bifurcation diagrams in the (*h*_A_, *Ca*, *V*)-space (in a manner similar to Fig 11) to determine how [Ca^2+^]_*i*_ affects pseudo-plateau bursting dynamics, we obtain the critical manifold shown in Fig 13**C** (gray surface). Superimposing the BPO in panel **B** on the critical manifold in **C** shows that the persistent spiking during the active phase seen in the trajectory in Fig 11 is replaced by damped spiking that are triggered when a trajectory jumps from the lower stable sheet at the left fold defined by SN_1_ to the upper stable sheet. These damped oscillations spiral around the upper sheet of the stable equilibria until the trajectory passes through the curve of Hopf bifurcations HB (where a sheet or a family of envelopes of unstable POs—plotted as a green sheet—emanates from) without being repelled by the unstable sheet of equilibria to the right of the HB curve. This phenomenon is known as “a slow passage through a Hopf” or the “ramp”, previously studied in [61]. When the trajectory eventually reaches the right fold defined by SN_2_, it drops down again to the lower stable sheet, and the cycle repeats itself. This type of dynamics is a hallmark of pseudo-plateau bursting.

For further illustration of how pseudo-plateau burst cycles behave in parameter space, we repeat the same analysis performed in Fig 12 by plotting the bifurcation curves identified in Fig 13**B** and 13**D**. Superimposing a burst cycle (blue) on top of these curves shows how crossing the SN_1_ curve triggers damped spiking in the active phase while crossing the SN_2_ curve brings the trajectory back to the silent phase (Fig 13**D**).

**Fig 14:**
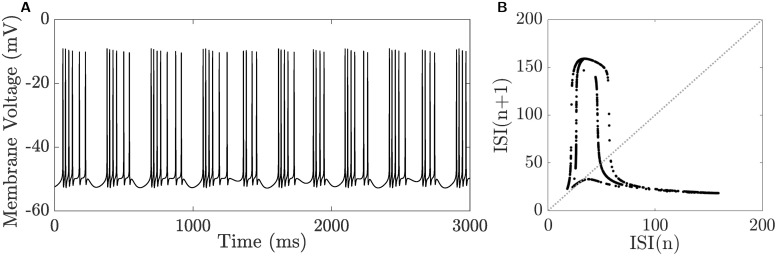
Chaotic bursting in the CSC model defined by the full system (1). **A**) Time course of the membrane voltage *V* when exhibiting chaotic bursting obtained by setting *g*_K(Ca)_ = 3 *μ*S.cm^−2^ and *g*_HVA_ = 0.215 *μ*S.cm^−2^. **B**) The return map of the interspike interval (ISI) for the time course of *V* in **A**.

One peculiar feature about the full system (1) is its ability to generate chaotic bursting [62–64] in which the number of spikes in its active phases are not uniform. Fig 14**A** shows an example of such a BPO when *g*_HVA_ = 0.215 mS.cm^−2^ and *g*_K(Ca)_ = 3 mS.cm^−2^. To further verify the irregularities of this BPO, we plot in Fig 14**B** the return map of its interspike interval (ISI). This generates patterns that are off the diagonal and similar to those produced by other chaotic spiking models [65, 66], suggesting that the BPO in Fig 14**A** is chaotic. The presence of such bursting activity implies that there are additional parameter regimes associated with new and distinct neuronal activity not previously captured by Fig 9.

### Regimes of behaviour associated with *g*_HVA_ and *g*_K(Ca)_

**Fig 15:**
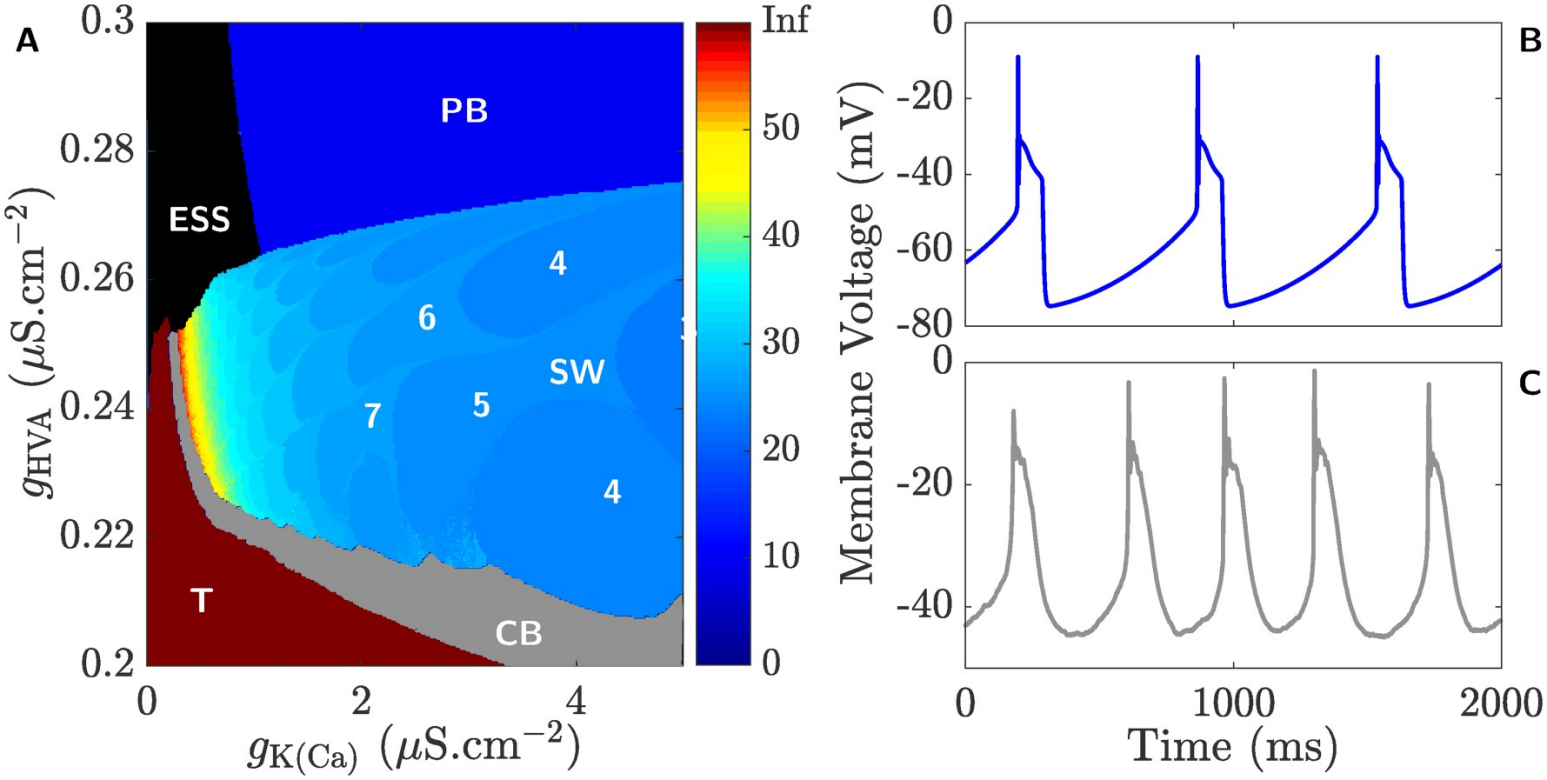
The effects of varying *g*_HVA_ on the dynamics of the full system (1). **A**) Color-map of the various regimes of behavior in the (*g*_K(Ca)_, *g*_HVA_)-plane when *g*_A_ = 10.2 *μ*S.cm^−2^. The square-wave bursting regime, labeled **SW**, is color-coded based on the number of spikes in their corresponding bursting orbits calibrated according to the color-bar to the right. Additional regimes are also present, including a tonic firing regime (labeled **T**), a pseudo-plateau bursting regime (labeled **PB**), a quiescent regime with elevated steady state (labeled **ESS** in black) and a chaotic bursting regime (labeled **CB** in gray). Since the full system (1) fires regularly in regimes **T** and **PB**, these regimes are also color-coded according to the color-bar to the right. For further clarity, the number of spikes in various regions of **SW** are displayed. **B**) Time course of the membrane voltage *V* showing pseudo-plateau bursting obtained by setting *g*_HVA_ = 0.77 *μ*S.cm^−2^, while keeping the other maximum current conductances at their default values listed in Table 2. **C**) Electrical recording of one CSC showing pseudo-plateau bursting activity induced by the application of 20 mM 4-AP in the absence of a hyperpolarizing step current.

Computing the loci of the SNP-PD curves in (*g*_K(CA)_, *g*_A_)-plane in Fig 9 proved to be very challenging and even more so when expanding this approach to the (*g*_K(CA)_, *g*_HVA_)-plane. To overcome this problem in the latter case, we utilize in Fig 15**A** another approach in which we apply a parameter sweeping method that initially computes the solution trajectories of the full system (1) for a specific set of maximum conductances listed in Table 2, followed by counting the number of spikes in each BPO [41, 67].

Based on this approach, we find that the square-wave bursting parameter regime (labeled again as **SW** in Fig 15**A**) is surrounded by four other regimes, labeled **T**, **ESS**, **PB** and **CB**. In the parameter regime **T** (burgundy), the full system (1) produces tonic firing activity identical to that seen in Fig 1, whereas in **ESS**, it exhibits quiescent activity in the form of an elevated steady state representing the depolarization block; in the parameter regime **PB**, it displays pseudo-plateau bursting that we alluded to when discussing the olive envelope of periodic orbits in Fig 5**A**, whereas in the regime **CB**, it exhibits chaotic bursting activity with no fixed number of spikes per burst similar to that seen in Fig 14**A**. Since periodic orbits in **T** are tonically firing, it is color-coded burgundy to indicate that the number of spikes per “burst” is infinite in this regime. Similarly, since trajectories in **ESS** and **CB** are quiescent or possess irregular number of spikes per burst, respectively, they have been assigned the neutral colors black and gray (that do not belong to the color-scale) to distinguish them from other regularly spiking regimes.

As suggested by Fig 15**A**, increasing *g*_HVA_, while keeping *g*_K(Ca)_ fixed, eventually shifts the dynamics of the full system (1) into the **PB** regime. Fig 15**B** displays a typical pseudo-plateau bursting solution from this regime, reminiscent to that seen in Fig 13**A**. We have shown that a low dose of 4-AP potentiates *I*_HVA_ and partially blocks *I*_A_ and *I*_K_. Thus to test the prediction that CSCs turn into pseudo-plateau bursters when *g*_HVA_ is increased significantly, one can apply higher doses of 4-AP (∼20 mM). Our results reveal that by doing so, 4 out of 5 CSCs produce pseudo-plateau bursting activity identical to the recording shown in Fig 15**C**, confirming our prediction. Interestingly, a hyperpolarizing step current (ranging between -6 and -17 pA) is applied to 3 out of the 4 pseudo-plateau bursting CSCs to generate such bursting pattern, an expected requirement in view of the fact that 4-AP partially blocks *I*_A_ and *I*_K_, the two outward repolarizing currents. This suggests that the hyperpolarizing step current is needed to overcome the effect of 4-AP on *I*_A_ and *I*_K_. It should be mentioned here that the spiking profile of the outlier cell has also a short elevated plateau segment following each spike (results not shown). This seems to suggest that this specific cell is also a pseudo-plateau burster, but lacks the ability to generate multiple spikes per burst because its fold and subcritical Hopf bifurcations, highlighted in Fig 13**C**, are closer to one another in this case compared to those associated with other cells.

The results obtained here thus demonstrate that there are many regimes of behavior that are not only restricted to burst firing, with peculiar irregularities in their spike patterns (like the square-wave burster), but also to other behaviors that are not actually related to bursting.

## Discussion

Cerebellar stellate cells (CSCs) are spontaneously active neurons that tonically fire action potentials in the absence of synaptic inputs [11]. These neurons, however, appear to be also capable of bursting when stimulated by pharmacological agents that can block or potentiate specific ionic currents. We have shown in this study, through patch clamping, that (i) the application of low doses of 4-Aminopyridine (4-AP), known to partially block *I*_K_ and *I*_A_, induces a specific mode of bursting in CSCs whose active phase duration may vary significantly between neurons, and that (ii) the application of low doses of 4-AP in combination with cadmium (Cd^2+^), a known blocker of *I*_HVA_, can induce another mode of bursting with active phases that have no spiking towards the end of the phase. This seems to suggest that CSCs have the machinery to produce these two modes of bursting, and that the maximum conductances of *I*_K_ and *I*_A_, on one hand, and *I*_HVA_, on the other, are key players in producing them. What the implications are of such bursting activities on cerebellar physiology and on development remain unclear. In this study, we applied modeling and computational techniques to investigate how 4-AP and Cd^2+^ affect the electrical properties of CSCs and to analyze dynamically the two modes of bursting detected.

By expanding a Hodgkin–Huxley type model, previously developed to describe tonic firing in CSCs, we were able to replicate the main electrophysiological features of isolated and spontaneously active CSCs, including tonic spiking, runup, non-monotonic first-spike latency and finally switching in responsiveness. The originally revised Hodgkin–Huxley model presented in [16] was expanded through the inclusion of two key ionic currents *I*_K(Ca)_ and *I*_HVA_ as well as the inclusion of a flux-balance equation that describes the dynamics of [Ca^2+^]_*i*_. The new model was able to capture the two modes of bursting activities that these neurons exhibit under two pharmacological conditions: namely, when bathed in 2 mM 4-AP only, or bathed in 2 mM 4-AP and 300 *μ*M Cd^2+^. The inclusion of Ca^2+^ into the model was crucial for inducing bursting as it added one slow variable to the dynamics. Furthermore, a result of adding these new components to the model, parameter values had to be adjusted in order to still capture all the features of CSCs (see Tables 1 and 2).

The way that we were able to replicate bursting activities in CSCs seemed quite counter-intuitive as a low dose of 4-AP was applied as a channel blocker that inhibits *I*_A_ and *I*_K_. However, recent experimental evidence suggests that 2 mM 4-AP can induce threefold increase in HVA Ca^2+^ currents [52, 54] while it only blocks A-type potassium currents by at most 20% [18]. The broad heterogeneity that exists between CSCs in the way they respond to 4-AP, with active phases that have large variations in the number of spikes, suggests that the effects of small differences in the kinetics of *I*_HVA_ and *I*_A_ (including maximum conductances) between neurons can be amplified by the application of 4-AP. Model simulations showed that this was in fact the case, with small variations in the maximum conductances of these ionic currents, along with *I*_K(Ca)_, were able to induce significant changes in the number of spikes in the active phases in bursting periodic orbits (BPOs).

To demonstrate this, we provided a bifurcation analysis of the model by targeting the relevant maximum conductances that were affected by the application of 4-AP. More specifically, we computed the bifurcation diagram of the system with respect to *g*_HVA_, *g*_A_ and *g*_K(Ca)_. These bifurcation diagrams demonstrated that the bursting activities of these neurons are generated by a family of isolas, and that these isolas are not necessarily organized in a sequential order in which the difference between the number of spikes in adjacent ones is one. This new phenomenon that we termed non-sequential spike adding, produced wide range of differences in the number of spikes between BPOs produced by small variations in the maximum conductances of the three ionic currents specified. This sensitivity to parameter variations is a manifestation of the effect of 4-AP in amplifying differences in responses between neurons.

By applying slow-fast analysis, treating *h*_A_ and *Ca* as parameters, we were able to explain the underlying mechanisms of bursting activities. Our results revealed that this type of bursting is square-wave (fold/homoclinic) with one unique feature that the envelope of stable periodic orbits responsible for the active phase of the burst belongs to the outer branch of an isola that eventually loses stability at a period-doubling bifurcation. Because *h*_A_ was not slow enough, the trajectories were able to skip the period doubling bifurcation points and terminate their active phases at a homoclinic bifurcation. We conjectured, however, that the existence of a cascade of such period-doubling bifurcations of the fast subsystem leads to such a non-sequential spike adding. To confirm this conjecture the system should be further investigated by continuing the period-doubling bifurcation points in parameter space and finding out how these curves govern dynamics.

When applying slow-fast analysis in this study, we used two distinct time scales, slow and fast. Given the existence of three different time scales in the full system (1), it would have been more appropriate to analyze it by decomposing it into three subsystems. This could have more precisely explained the non-sequential spike-adding process in square-wave bursting. Moreover, considering the previous studies carried out on ‘sequential’ spike-adding phenomena, it is very likely that canards might also play a role not only in the non-sequential spike-adding process, but also in generating chaotic bursting activity. The role of canards in governing dynamics is evidenced in the presence of after-depolarizations (ADPs) in Fig 15**A**, where trajectories of BPOs are likely traveling briefly along the unstable sheets of the critical manifold shown in Figs 11 and 13**C**, producing these ADPs. Validating the involvement of three time scales and canards in these processes represents a new avenue for future research.

One interesting feature we discovered about the model is that there are tiny intervals of bistability between the isolas with different number of spikes when plotting the bifurcation diagram of the membrane voltage *V* with respect to HVA maximum conductance. These intervals are confined by period-doubling and saddle-node bifurcations of periodic orbits. By continuing these bifurcations in two-parameter space, involving *g*_A_ and *g*_K(Ca)_, we were able to determine boundaries between different parameter regimes, each possessing a specific number of spikes. We also explained how some of these isolas appear and disappear in parameter space based on these boundaries. The results thus suggest that the number of spikes is sensitive to variations in maximum current conductances.

In this study, we have also demonstrated that variations in *g*_A_ does not affect the type of bursting and only alters the number of spikes in each burst. By varying the maximum conductances of *I*_HVA_ and *I*_K(Ca)_ simultaneously, the model was able to produce other distinct regimes of behavior not previously observed in the other parameter space defined by *g*_K(Ca)_ and *g*_A_. That included the pseudo-plateau bursting regime and the chaotic bursting regime. The pseudo-plateau bursting was obtained by increasing *g*_HVA_ significantly while keeping *g*_K(Ca)_ fixed, a prediction that was confirmed experimentally by applying 20 mM 4-AP, a potentiator of *I*_HVA_ as demonstrated in this study. Hyperpolarization step currents were applied in most of these recordings to overcome the partial blockage of *I*_A_ and *I*_K_ by 4-AP.

At high *g*_K(Ca)_ and low *g*_HVA_ and *g*_K_, the full system (1) also exhibited pseudo-plateau bursting. Slow fast analysis of this type of bursting revealed that the bifurcation diagram of the membrane voltage *V* with respect to the slow variable *h*_A_ exhibited an almost identical configuration to the one produced by the square-wave bursting model, except for the absence of the isolated envelope responsible for spiking in the active phase of the latter burster. Spiking in the active phase of the pseudo-plateau burster was generated by the damped spiking around the upper stable branch of the Z-shaped curve, followed by a non-spiking segment when trajectories passed the delayed Hopf, generating the ramp effect (hence the name fold/sub-Hopf).

One interesting aspect of pseudo-plateau bursting is that electrical recordings of CSCs bathed in 2 mM 4-AP and 300 *μ*M Cd^2+^ induced very similar type of bursting activity. Considering the location of the parameter regime that produced this bursting, it seems that Cd^2+^ is reducing the 4-AP-dependent *I*_HVA_ potentiation while simultaneously causing *I*_K(Ca)_ to potentiate. Evidence of the latter has been documented in previous studies [60, 68], demonstrating that Cd^2+^ can enter the cell through Ca^2+^ channels (in this case, though T-type Ca^2+^ channels included in the full system (1)) and then act as a Ca^2+^ agonist capable of activating K(Ca) channels. This means that striking the right balance between the maximum conductances of these two currents, along with 4-AP-sensitive *I*_K_ and *I*_A_ maximum conductances, can produce such bursting profile.

One interesting aspect of the model presented in this study is its ability to exhibit a chaotic bursting behavior. Indeed, by using a return map of ISI, we discovered that in this bursting pattern, the model neuron fires noise-independent, yet irregular bursts of action potentials whose active and silent phases are non-uniform. This suggests that there are other mechanisms, such as the presence of multiple time scales and canards, that are in play when it comes to generating such irregular activity. Analysis of this type of bursting and its underlying mechanism(s) is left for future studies.

Although the two modes of bursting activities highlighted in this study (i.e., the square-wave and pseudo-plateau bursting) are pharmacologically induced, it shows that CSCs possess all the necessary ingredients to exhibit such rhythmic activities. The fact that CSCs are inhibitory interneurons that synapse onto Purkinje cells, the main output of the cerebellum, suggest that these ingredients may be necessary under certain conditions (e.g., during development). Demonstrating their ability to induce bursting in vivo and showing how they could be triggered, remain to be seen.

## Supporting information

S1 Text Straightforward definitions of all concepts adopted from the field of dynamical systems. (PDF)
