# Supplementary material for: Bursting in cerebellar stellate cells induced by pharmacological agents: Non-sequential spike adding
Source: PLoS Comput Biol. 2020 Dec 14;16(12):e1008463. doi: 10.1371/journal.pcbi.1008463 (PMC7769625; doi:10.1371/journal.pcbi.1008463)
Supplement: S1 Text — (PDF) [file pcbi.1008463.s001.pdf]

List of Dynamical Systems Concepts

Saeed Farjami[1], Ryan P. D. Alexander[2],
Derek Bowie[2] and Anmar Khadra[1]
Department of Physiology[1]
Department of Pharmacology and Therapeutics[2]
McGill University, Montréal, QC, Canada

Here we provide straightforward definitions of all concepts adopted from the field of dynamical systems [1,2] used in this study. Let $\dot{\mathbf{X}} = \mathbf{f}(\mathbf{X}; \Lambda)$ be an autonomous system of ordinary differential equations that depends on the parameter Λ.

- Phase space: is the space defined by the state variables of the system.
- Equilibrium (also called fixed point or steady state): is the solution to the equation $\mathbf{f}(\mathbf{X}; \Lambda) = 0$. An equilibrium is labeled stable/unstable if a small perturbation away from this equilibrium eventually converges/diverges to/away from it, respectively (i.e., it acts as an attractor/repeller). If the equilibrium is labeled a saddle, then it is not an attractor or repeller. A saddle can have a stable and an unstable manifolds.
- Saddle-node: is a bifurcation point that occurs at a specific value of Λ where a stable and an unstable equilibria merge.
- Periodic orbit (also called limit cycle): is a solution to the system that oscillates in time. It could be attracting, repelling or of saddle type.
- Bursting periodic orbit: is a periodic orbit that alternates between an active phase, comprised of a cluster of action potentials, and a silent phase.

- Hopf bifurcation: is a bifurcation point that occurs at a specific value of Λ where the stability of the equilibrium changes and periodic orbits emerge.

- Homoclinic bifurcation: is a a bifurcation point that occurs at a specific value of Λ when a periodic orbit merges with the stable and unstable manifolds of a saddle fixed point (i.e., the period of the limit cycle becomes infinite).

- Saddle-node on an invariant cycle (SNIC): is a bifurcation point that occurs at a specific value of Λ where a homoclinic bifurcation meets at a saddle-node bifurcation.

- Isola: is a family of equilibria/periodic orbits detached from the main bifurcation diagram; it usually appears and disappears via saddle-node bifurcations.

- Slow-fast system: is a multidimensional dynamical system defined by $\mathbf{X}$, whose variables evolve at either fast or slow time scales. It can be divided into two subsystems, a slow and a fast one. By assuming that the slow variables are parameters, the dynamics of the fast subsystem (also called the reduced system) can be analyzed accordingly.

- Critical manifold: is the family of equilibria of the reduced system when the slow variables are treated as parameters; it could be a high-dimensional structure if the number of slow variables (treated as parameters) is at least two. The equilibria could be stable and/or unstable, generating attracting and repelling sheets (or branches in the case of 1 slow variable).

- Canard: is a special solution of a slow-fast system that follows the unstable sheet of the critical manifold.

References

[1] Strogatz SH. Nonlinear dynamics and chaos: with applications to physics, biology, chemistry, and engineering. New York: Avalon Publishing; 2014.

[2] Kuznetsov Y. Elements of Applied Bifurcation Theory. New York: Springer; 2004.
